# Ag/ZnO core–shell NPs boost photosynthesis and growth rate in wheat seedlings under simulated full sun spectrum

**DOI:** 10.1038/s41598-023-41575-7

**Published:** 2023-09-01

**Authors:** Shahnoush Nayeri, Mahboubeh Dolatyari, Neda Mouladoost, Saeed Nayeri, Armin Zarghami, Hamit Mirtagioglu, Ali Rostami

**Affiliations:** 1 SP-EPT Lab., ASEPE Company, Industrial Park of Advanced Technologies, Tabriz, Iran; 2 Photonics and Nanocrystal Research Lab. (PNRL), Faculty of Electrical and Computer Engineering, University of Tabriz, Tabriz, 51666 Iran; 3 Department of Statistics, Faculty of Science and Literature, University of Bitlis Eren, Bitlis, Turkey

**Keywords:** Biotechnology, Physiology, Plant sciences, Materials science, Nanoscience and technology, Optics and photonics, Physics

## Abstract

Breeding programs rely on light wavelength, intensity, and photoperiod for rapid success. In this study, we investigated the ability of Ag/ZnO nanoparticles (NPs) to improve the photosynthesis and growth of wheat under simulated full solar spectrum conditions. The world population is increasing rapidly, it is necessary to increase the number of crops in order to ensure the world’s food security. Conventional breeding is time-consuming and expensive, so new techniques such as rapid breeding are needed. Rapid breeding shows promise in increasing crop yields by controlling photoperiod and environmental factors in growth regulators. However, achieving optimum growth and photosynthesis rates is still a challenge. Here, we used various methods to evaluate the effects of Ag/ZnO NPs on rice seeds. Using bioinformatics simulations, we evaluated the light-harvesting efficiency of chlorophyll *a* in the presence of Ag/ZnO NPs. Chemically synthesized Ag/ZnO nanoparticles were applied to rice grains at different concentrations (0–50 mg/L) and subjected to a 12-h preparation time. Evaluation of seed germination rate and growth response in different light conditions using a Light Emitting Diode (LED) growth chamber that simulates a rapid growth system. The analysis showed that the surface plasmon resonance of Ag/ZnO NPs increased 38-fold, resulting in a 160-fold increase in the light absorption capacity of chlorophyll. These estimates are supported by experimental results showing an 18% increase in the yield of rice seeds treated with 15 mg/L Ag/ZnO NPs. More importantly, the treated crops showed a 2.5-fold increase in growth and a 1.4-fold increase in chlorophyll content under the simulated full sun spectrum (4500 lx) and a 16-h light/8-h dark photoperiod. More importantly, these effects are achieved without oxidative or lipid peroxidative damage. Our findings offer a good idea to increase crop growth by improving photosynthesis using Ag/ZnO nanoparticle mixture. To develop this approach, future research should go towards optimizing nanoparticles, investigating the long-term effects, and exploring the applicability of this process in many products. The inclusion of Ag/ZnO NPs in rapid breeding programs has the potential to transform crops by reducing production and increasing agricultural productivity.

## Introduction

### Background and importance

Bread wheat (*Triticum* *aestivum*) is an important staple food grown on approximately 217 million hectares of land with a world production of approximately 760 million tons (FAO, 2022). Rapid population growth (DESA, United Nations), expected to reach 9.7 billion in 2050 and over 11 billion in 2100, poses an urgent challenge to sustainable food production. Meeting nutritional and care needs requires new cultivation methods to produce high-quality, sustainable seeds. However, traditional breeding programs require a longer recovery or breeding period, including 4 to 6 generations with only 1–2 generations per year^1,2^. Addressing these limitations requires technologies that reduce energy production and increase crop yields and food security.

### Rapid breeding and photoperiod modulation

In this context, Rapid Breeding (SB) technology has emerged as an effective means of shortening the crop cycle by controlling photoperiod, photoperiod, and other environmental factors in growth control^1^. The results were successful, with the growth cycle extended to 6 generations per year for wheat, barley, chickpea, pea, Brachypodium distachyon, and to 4 generations per year for canola^2,3^. Previous studies have reported results using a photoperiod of 22 h of high-energy LED (photosynthetically active radiation, PAR, 400–700 nm, use 360–650 µmol/m^2^s^–1^) after 2 pm, support. Early flowering and long-day harvested crops^2–4^. Extending this concept to diurnal and short-day facilities exhibiting independent photoperiod or short solar criticality remains challenging^5,6^. In addition, the optimum use of light and temperature in the rapid expansion chamber is still a challenge in the development of this process.

### Role of nanomaterials in improving photosynthesis

Nanomaterials have received great attention for their many uses, particularly in improving photosynthesis and plant growth. Light collection systems based on metal nanoparticles have shown great potential in photosynthetic photosynthesis, energy production, photocatalysis, solar cells, and biosensing^7–12^. The light enhancement obtained by surface plasmon resonance (LSPR) in metallic nanoparticles (Ag, Au, Pt) has received great attention^13^. This effect was observed in studies where the fluorescence emission of photosynthetic pigment-protein light-harvesting 2 (LH2) complexes increased upon excitation at Au plasmon resonance^14–16^. The plasmon resonance of Ag NPs shows enhanced fluorescence in cyanobacterial PSI complexes, demonstrating their ability to enhance natural photosynthesis^17^. Photocatalytically active metal nanoparticles containing TiO_2_, ZnO, CuO, and SiO_2_ NPs increase photosynthesis and plant growth by effectively affecting chlorophyll *a* and photosystem complex elements^18–26^. In addition, metal nanoparticles are recognized as effective nano fertilizers, nano pesticides, nano fungicides, and nano herbicides to improve plant resistance to various environmental stresses. Recent studies have highlighted the potential of NPs, particularly iron oxide NPs, in promoting growth in various crops. These NPs have shown concentration-specific effects in Capsicum annuum plants, reorganizing leaf structures and enhancing chloroplast stacking^27^. Similar benefits have been observed in green gram sprouts, spinach, and other species^28–31^. Moreover, NPs have proven to be effective substitutes for traditional fertilizers, boosting yields in crops like chili, marigold, and rice^30,31^. However, it is important to consider the environmental impact of direct NP application, as excess amounts can induce stress responses and amino acid reductions in plants^32^. This has prompted the exploration of novel strategies, such as the seed presoak approach using NPs, to optimize plant growth while minimizing environmental impact^33^. overall, NPs hold significant potential for revolutionizing agriculture, as demonstrated in studies involving crops like green pepper, green beans, and pomegranates^30–34^. These insights underscore the broader role of NPs in accelerating crop growth and development, aligning with the concept of speed breeding and advancing sustainable agricultural practices.

### Ag/ZnO nanoparticles: a new light harvest antenna

Among nanomaterials, silver/zinc oxide nanoparticles (Ag/ZnO NPs) appear as efficient nanoparticles. Zinc oxide nanoparticles (ZnO-NPs) are recognized as a biocompatible material for organisms^35,36^. Previous investigations have highlighted the potential of ZnO-NPs in stimulating seed germination and promoting plant growth. Additionally, their antimicrobial properties contribute to disease suppression and plant protection1. Studies in the literature reveal compelling outcomes: ZnO NPs have shown a significant augmentation in various physical parameters of wheat when compared to control conditions, particularly under salt stress. The application of ZnO NPs resulted in substantial improvements, including a 24.6% increase in chlorophyll *a* and *b* contents, a 34.6% enhancement in plant height during both vegetative and maturity stages, a 30.7% boost in shoot length, a 27.6% increase in spike length, a remarkable 74.5% elevation in root fresh weight, a notable 63.1% increment in root dry weight, and an impressive 42.2% rise in wheat grain yield^37–42^. Silver nanoparticles exhibit distinctive optical properties attributed to resonance effects originating from the presence of conduction electrons within their structure. Upon interaction with photons, these electrons give rise to the localization of electric fields at interfaces with the surrounding environment. Regulatory effects of Ag NPs on genes related to iron, plant diseases, oxidative stress, and hormonal stimuli have been observed in Arabidopsis^41,42^.

### Research aims and methods

This study investigated in depth the improvement of light quality and photosynthesis rate of chlorophyll *a* in the presence of Ag/ZnO nanoparticles. Ag/ZnO NPs possess unique optical properties, including localized surface plasmon resonance (LSPR), which can lead to enhanced light absorption. This property makes them suitable for serving as efficient artificial light-harvesting antennas, thus potentially boosting photosynthesis and growth rates in plants. The choice of Ag/ZnO NPs also is influenced by their stability and biosafety. Ensuring that nanoparticles are biocompatible and do not harm the environment or the plants is crucial. According to prior studies suggesting the positive effects of Ag and ZnO NPs on plant growth, photosynthesis, and stress resistance. We investigated the effects of different concentrations (0–50 mg/L) of chemically synthesized spherical Ag/ZnO composite nanoparticles on seed germination and plant growth of rice grains using bioinformatics methods. These experiments were performed under different lighting conditions simulated in an LED-based grow room followed by rapid growth.

## Materials and methods

### Theoretical modeling of the effect of Ag/ZnO NPs on chlorophyll a molecule

The finite element analysis approach is used to predict the optimal size of Ag/ZnO spherical nanoparticles to realize an efficient photosynthesis rate. To spectrally overlap the Surface Plasmon Resonance (SPR) with the maximum absorption of the dye, electric field profile, optical response, and size of the nanoparticles have been tuned using the wave optic module of COMSOL Multiphysics. The dielectric function of bulk Ag/ZnO was extracted from experimental data provided by Johnson and Christy^43^.

### Theoretical analysis of Ag/ZnO nanoparticle extinction, scattering, and absorption cross-sections

Here, we have investigated the scattering of an incoming electromagnetic wave by a spherical particle using COMSOL Multiphysics and the results of this study are compared with the analytical results of the Mie solution. The strategy employed to enhance photosynthetic efficiency is to increase the light absorption of the pigment molecule by manipulating the plasmonic effect of metallic nanoparticles. Here, Ag/ZnO nanoparticles with 30 nm in diameter were chosen possessing LSPRs in the spectral range of 350 to 450 nm. To overlap the extinction frequency band of the NPs with the maximum absorption of the pigment molecule, the optimal size has been calculated by finite element simulations. The nanoparticle is embedded in a non-absorbing medium and excited by a plane wave with an amplitude of E_0_ at $\lambda =500 \mathrm{nm}$. The Maxwell equation (Eq. 1) is used for the scattered electric fields measurement as follows,

1 $$ \nabla \times \left( {\frac{1}{{\mu_{r} }}\nabla \times E_{sca} } \right) - k_{0}^{2} \left( {\varepsilon_{r} - j\frac{\sigma }{{\omega \varepsilon_{0} }}} \right)E_{sca} = 0, $$

where ${\varepsilon }_{r}$ and ${\mu }_{r}$ are the relative permittivity and permeability, $\sigma$ is the conductivity representing the losses, and $\omega$ is the angular frequency^44^.

### Calculation of optical properties of the chlorophyll a molecule

Here, the time-dependent density functional theory (TDDFT) calculations were performed to determine the absorption peaks of chlorophyll* a* molecule. This fundamental step is required to find the spectral overlap between the calculated extinction of nanoparticles and light absorption by the pigment, and hence, improve the absorption through the plasmonic effects in the spectral range of 300 to 800 nm (Fig. S1). The initial model of the Mg porphyrin molecule was retrieved from PubChem (https://pubchem.ncbi.nlm.nih.gov/compound/Magnesium-Porphine). To add the chlorophyll molecule as a new substance to the Comsol material library, we require the optical properties of the pigment. As the real part of its refractive index (n) is equal to 1.52^45^, we have to determine the wavelength-dependent imaginary part of the refractive index in the visible spectrum. Using Bouguer-Beer-Lambert Law^46^, the absorption was calculated using the molar extinction coefficient (Eq. 2) at the spectral range of interest as,

2 $$ \log_{10} (\frac{{I_{0} }}{I}) = A = \varepsilon .\,L.\,c, $$

where e is the molar extinction coefficient [L/mol.cm], L is the diameter of the Cuvette and the distance that light travels [cm], c is the concentration of the desired substance [mol/L], I_0_ is the initial light intensity, and I is the light intensity after passing the diameter of the Cuvette. Assuming that the diameter of the Cuvette is 1[cm] and the concentration of chlorophyll is 1 [mol/L], the absorption coefficient can be estimated by Eq. (3),

3 $$ \alpha = \frac{1}{d}\log_{10} \left( {\frac{I}{{I_{0} }}} \right). $$

The complex refractive index of the chlorophyll *a* molecule was extracted in the spectral range of 300 to 800 nm.

### Materials

All reagents were analytic grade and purchased from Sigma-Aldrich, Germany.

0.024 g silver acetate, and 0.6 g polyvinyl pyrrolidone (PVP), were dissolved in 450 ml deionized water and stirred for 5 min. Then, 0.1 g sodium borohydride was dissolved in 50 ml deionized water, drop wised inserted into the first solution, and stirred for 30 min. 0.11 g Zinc acetate dehydrate was added to the solution and 0.1 g sodium borohydride was dissolved in 500 ml deionized water and the drop wised inserted into the solution again. The resulting nanoparticles were centrifuged and washed with water and absolute alcohol 3 times. The resulting particles are dispersed in water.

### Transmission electron microscopy (TEM) and energy-dispersive spectroscopy (EDX) analysis

The size and shape of Ag/ZnO composite NPs were measured at × 200–× 300 magnifications using TEM microscopy imaged by a Zeiss-EM10C TEM microscope at 80 kV accelerating voltage and ~ 2 nA beam current. The EDX analysis was employed to identify the elemental composition of the Ag/ZnO composite NPs.

### UV–visible spectroscopy

UV–Vis absorption spectra were recorded employing a PG Instruments Ltd T70 UV/Vis spectrophotometer.

### Dynamic light scattering (DLS) and zeta-potential analysis

The hydrodynamic particle size analysis was performed by dynamic light scattering (DLS) using Nanotrac Wave II Q** (**Microtrac MRB Co., Pennsylvania, USA).

### Plant material and surface sterilization

The winter bread wheat seeds (*Triticum aestivum* L. *var.* OmidBakhsh; genotype CD-98) were purchased from Agricultural Research, Education and Extension Organization (AREEO), Tabriz, Iran. All plant experiments were carried out in accordance with relevant guidelines. The seeds were surfaced and sterilized using 20% commercial bleach solution (Sodium Hypochlorite with 5% active chlorine) for 3 min and rinsed three times for 2 min each with distilled deionized water.

### The effects of NPs concentrations and priming time on seed germination

To investigate the effect of Ag/ZnO composite NPs on seed germination rate, the wheat seeds were soaked in the Ag/ZnO NPs colloids in five different concentrations of 0, 5, 15, 25, 50 mg/L for four durations of 6, 12, 18 and 24 h with continuous agitation at 22 °C in darkness condition. The primed seeds were rinsed three times with distilled deionized water for 3 min each and gently dried at room temperature to obtain natural moisture content. Deionized water was used for hydropriming as a control sample. The experiments were performed in a complete randomized design (CRD) in three replicates containing 50 seeds per replicate (petri dish). The germination percentage was defined as the percentage of the germinated seeds with 2 mm radical emergence on the 2nd day (48 h) in each experiment at 22 °C in dark conditions^47^. After 5 days of germination,10 seedlings were randomly selected on each plate to measure shoot and root length and their fresh and dry weight^48,49^. Seedling vigor indexes were calculated as described by Sunita & et al.^50^.

### LED-supplemented benchtop speed breeder box setup

**Figure 1:**
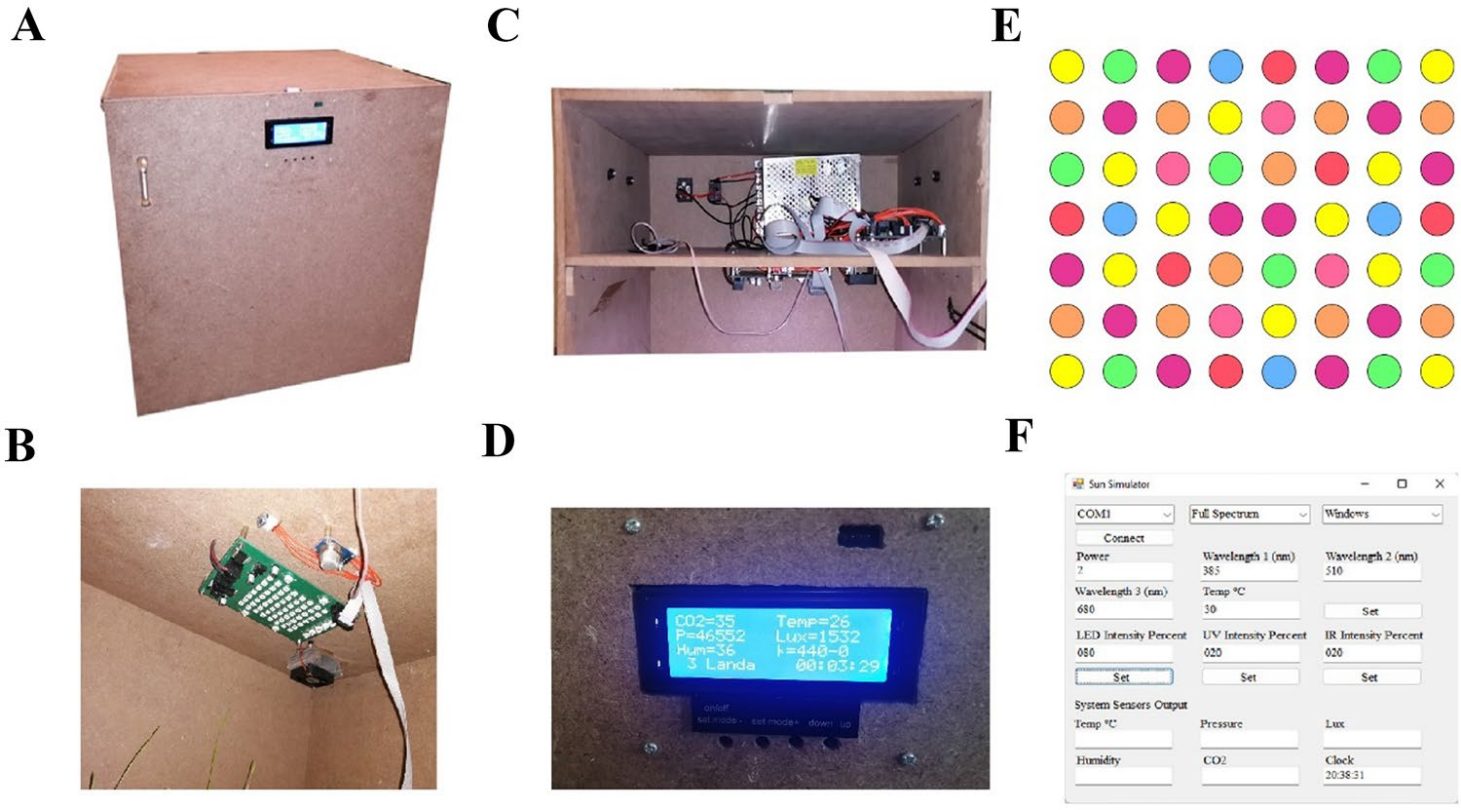
The structure of the automatic speed breeder chamber simulating the full solar spectrum and wide range of light wavelength. (**A**) Device structure; (**B**) The LED lightening PCB board, automatic fan, and barometer module in the top central position of the growth chamber; (**C**) The electronic system containing the mainboard and 12 V 15A power adaptor; (**D**) The LCD screen representing CO_2_ intensity, temperature (ºC), pressure (bar), light intensity (Lux), Humidity (%), wavelength range (λ), local time (hh:mm: ss); (**E**) Full RGB Pattern represented by 56 RGB LEDs; (**F**) The user interface of the windows application.

To evaluate the plant morphophysiological properties in different light conditions and Ag/ZnO NPs concentrations, we used the automatic RGB LED-supplemented benchtop speed breeder box (50 cm × 50 cm × 90 cm) providing a wide range of specific wavelengths in the light intensity of interest (Fig. 1). The outer and inner structures of the speed breeder chamber was shown in Fig. 1A and B, respectively. The electronic system was shown in Fig. 1C. It is adjustable using a user-friendly LCD screen and Windows software (Fig. 1D and F). The LED panel cover 22 peak wavelength in the range of 245–940 nm at 0.2 sun intensity compared with AM1.5G global solar spectrum sunlight containing 4 UV LED (245 nm), 56 RGB LED with a 10 mm center-to-center distance from each other (14 different peaks with 22 nm intervals in the range of 380–680 nm), five IR LEDs with different wavelengths, including 730, 740, 810, 850, and 940 nm in the area of 10 × 15 cm^2^ (Fig. 1E). The photoperiod, humidity, and temperature are also programable in the speed breeder box. The block diagram of the system operation was illustrated in Fig. S2.

### Plant growth condition

All plant experiments were carried out in accordance with relevant guidelines. The germinated seeds with a high germination rate (duration of priming treatment was 12 h) were selected for further wheat morphophysiological analysis in seven different light condition setups, including natural light, simulated full sun spectrum, 100% blue light (470 nm), 100% red (663 nm), 75% blue: 25% red, 50% blue: 50% red, and 25% blue: red 75%. The effect of five different concentrations of Ag/ZnO NPs (0, 5, 15, 25, and 50 mg/L) on the morphophysiological properties of wheat seedlings were independently examined in each light condition setup using soil application. The seedlings with the shoot and root 1 cm long each were sown in polyethylene pots (10 cm × 10 cm × 15 cm) containing the greenhouse soil. The greenhouse soil was collected from 0 to 30 cm depth of the surface layer, air-dried, and sieved using 2 mm mesh to remove wood chips and clots. The background Zn and Ag concentrations in the soil were 6.54 mg/kg and 0.001 mg/kg, respectively. The soil was classified as sandy loam soil containing 72% sand, 16% silt, and 12% clay with a pH of 7.61, and EC of 5.05 dS/m. The soil physicochemical properties are 2.53% organic carbon (OC), 4.36% organic material (OM), 0.26% total N, 135.63 mg/Kg available P, 1195.01 mg/Kg available K, 7.73 mg/Kg available Fe, 1.07 mg/Kg available Mn, and 1.04 mg/Kg available Cu. The planted pots were maintained at 24 °C, 16/8 h light/dark photoperiod exposing to 4500 Lux light intensity from RGB LED lamps. The first soil application of Ag/ZnO NPs suspensions (5 ml per pot) was performed on the third day after seed germination from the soil. The second and third soil applications were conducted at 7 days intervals. The plants were irrigated 2 days a week (5 ml per pot) with deionized water (pH = 7). To avoid spraying the soil, the pots were covered with aluminum foil. The experiments were performed in CRD design in three replicates containing 5 seeds per replicate (pot). The methodology proposed by Abdul-Baki and Anderson^51^ was employed. The vigor index was computed utilizing the formula: Seed Vigor Index = Germination% × (root length + shoot length).

### Plant morphological measurements

The morphological properties, including the shoot length (SL), the shoot diameter (SD), root length (RL), root diameter (RD), number of leaves (NL), and roots (NR), shoot and root fresh weights (SFW and RFW), shoot and root dry weights (SDW and RDW), the SDW to SFW and RDW to RFW ratios, root-shoot biomass ratio (BR), crop growth rate (CGR) and relative growth rate (RGR)^46^ were measured in three biological replicates from 1-month-old control and the Ag/ZnO NPs-treated plants.

### Chlorophyll and carotenoid contents

Total chlorophyll was extracted from leaf samples (100 mg) of 1-month-old control and the Ag/ZnO NPs-treated plants using acetone 80% as described by Arnon et al.^52^. The absorbances of the extracts were spectrophotometrically measured at 470, 645, and 663 nm wavelengths, which were measured in three biological replicates. Each biological replicate was run in three technical replicates. The total chlorophyll, chlorophyll* a*, and *b,* and carotenoid contents were calculated as previously described by Lichtenthaler^53^.

### Total phenol content (TPC)

Total phenol content in 1-month-old control and Ag/ZnO NP-treated plants was measured using the Folin-Ciocalteu reagent method as described by Lin et al.^54^. The leaf content was extracted using 85%acidic methanol. The supernatants were separated by centrifugation at 20,000×*g* for 20 min. The Gallic acid was used as a reference standard calibration curve. The 30 µl acid–methanol extract (65 µg/ml) was mixed with 75 µl Folin-Ciocalteu reagent, neutralized with 225 µl sodium carbonate solution (20%, w/v), and diluted up to 2 ml total volume with deionized water. The reaction mixture was incubated at room temperature for 30 min. The blue color absorbance was spectrophotometrically measured at 765 nm. The total phenolic contents were determined from the linear equation of Y = 0.0711X–0.4944, R^2^ = 0.9845 as mg/g gallic acid equivalent (GAE) of dry extract. The absorbance measurements were performed in three biological replicates. Each biological replicate was read in three technical replicates.

### Total flavonoids content

Total flavonoid content in 1-month-old control and Ag/ZnO NPs-treated plants were measured using the colorimetry method as described by Chang et al.^55^. The 100 µl acid–methanol extract (65 µg/ml) was mixed with 40 µl AlCl_3_.6H_2_O 10% w/v, 40 µl potassium acetate 1 M, 600 µl methanol 95% and diluted up to 2 ml total volume with deionized water. The quercetin (50–400 µg/ml) was used as a reference standard calibration curve. The reaction mixture was incubated at room temperature for 40 min. The blue color absorbance was spectrophotometrically measured at 415 nm. The total flavonoid contents were determined from the linear equation of Y = 0.083X–0.0043, R^2^ = 0.9979 as mg/g quercetin equivalent of dry extract. The absorbance measurements were performed in three biological replicates. Each biological replicate was read in three technical replicates.

### Lipid peroxidation analysis

Oxidative damage to leaf lipids was evaluated by measuring the malondialdehyde (MDA)content as described by Stewart et al.^56^. The 200 mg wheat leaf tissue was finely homogenized in 2 ml 3-chloro acetic acid (TCA) 0.1% using a pestle and mortar. The homogenate suspension was centrifugated at 13,500 rpm for 15 min. The 1 ml supernatant was added to 2 ml thiobarbituric acid (TBA) 0.5% (w/v) resolved in 20%TCA. The mixture was heated at 95 °C for 30 min and then chilled to 0 °C stopping the reaction. After centrifugation of the mixture at 8500 rpm for 10 min, the optical density of the supernatant was spectrophotometrically measured at 532 nm and 600 nm wavelengths. The content of MDA (nmol g^–1^ FW) was calculated using Eq. (4):

4 $$ {\text{MDA }}\left( {{\text{nmol g}}^{{ - {1}}} {\text{FW}}} \right) \, = \, \left[ {\left( {{\text{A532}} - {\text{A6}}00} \right) \, \times {\text{ V }} \times { 1}000/{\text{155 mM cm}}^{{ - { 1}}} } \right] \, \times {\text{ W}}, $$

V: the volume of crushing medium, W: the fresh weight of the leaf.

### Estimation of antioxidant enzymes activity

The 250 mg fresh leaf tissue from the control and the Ag/ZnO NPs-treated plants were finely homogenized using liquid nitrogen and dissolved in 100 mM sodium phosphate buffer (pH 7.4) containing 1% PVP, and 0.5% (v/v) Triton-X 100. After centrifugation of the homogenate at 4 °C at 20,000 rpm for 20 min, the supernatant was collected to determine antioxidant enzyme activities as the methods described by Jogeswar et al.^57^. To determine the superoxide dismutase (SOD) activity (Ug^−1^ protein), 100 μL enzyme extract was added to the solution containing 1 mL of 0.25 mM pyrogallol and 1.9 mL of 0.1 M sodium phosphate buffer (pH 7.4). The absorbance was spectrophotometrically measured at 420 nm^57,58^. To determine catalase (CAT) activity (Ug^−1^ protein), 100 μL enzyme extract was added to the solution containing 1 mL of 0.059 M H_2_O_2_ in 0.1 M sodium phosphate buffer (pH 7.4), and 1.9 mL of distilled water. The absorbance was spectrophotometrically measured at 240 nm^52^. The peroxidase (POD) activity (Ug^−1^ protein) of samples was determined spectrophotometrically at 470 nm as described by Zhang et al.^59^. Briefly, 40 μL enzyme extract was added to the solution containing 150 µl guaiacol 4% (v/v), 2.66 mL of 0.1 M sodium phosphate buffer (pH 7.0), 150 µl H_2_O_2_ 1% (v/v). The blank sample contained the same mixture without enzyme extract. The absorbance measurements were performed in three biological replicates. Each biological replicate was read in three technical replicates.

### Statistical analysis

The statistical analysis was performed using Microsoft Excel software 2019 software (Microsoft, Redmond, WA, USA) and IBM SPSS statistics ver.26.0 (SPSS Inc., Chicago, IL, USA). The results were analyzed using ANOVA, followed by the posthoc Tukey’s test for multiple comparisons in all experiments. The level of significance was set at 5%.

### Ethical statement

The authors confirm that experimental research on the wheat plant (*Triticum aestivum* L. *var*. OmidBakhsh; genotype CD-98), including the collection of plant material, complied with institutional, national, and international guidelines and legislation.

## Results and discussion

### Simulation results

#### Absorption studies of the chlorophyll a molecule and simulated results for absorption and scattering of the Ag/ZnO nanoparticles

**Figure 2:**
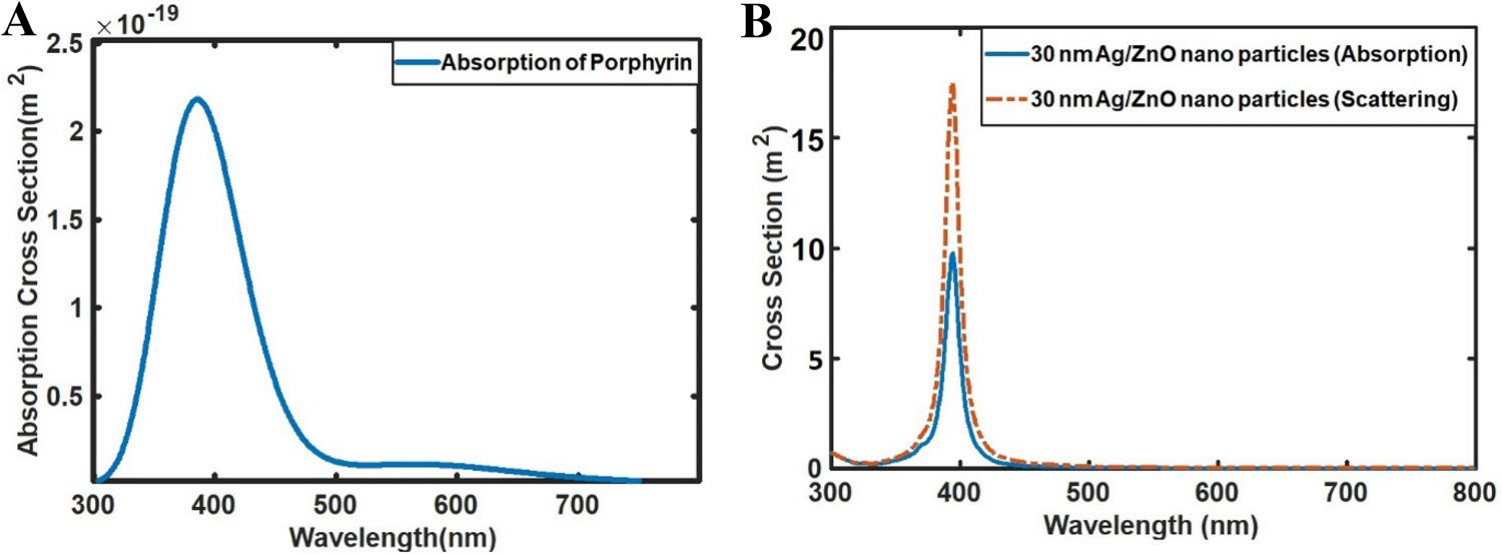
(**A**) The calculated Absorption spectra of a single chlorophyll molecule in an aqueous medium and (**B**) The calculated absorption and scattering of Ag/ZnO with a diameter of 30 nm.

Figure 2A and B exhibit the spectra related to absorption of chlorophyll-*a* molecules and simulated results for absorption and scattering of Ag/ZnO nanoparticles. As the figures show, the introduced nanoparticles can absorb and scatter the light with a wavelength of 394 nm and provide the extra light for the chlorophyll-*a* molecule.

#### Enhancement of the electric field by 30 nm Ag/ZnO nanoparticle

**Figure 3:**
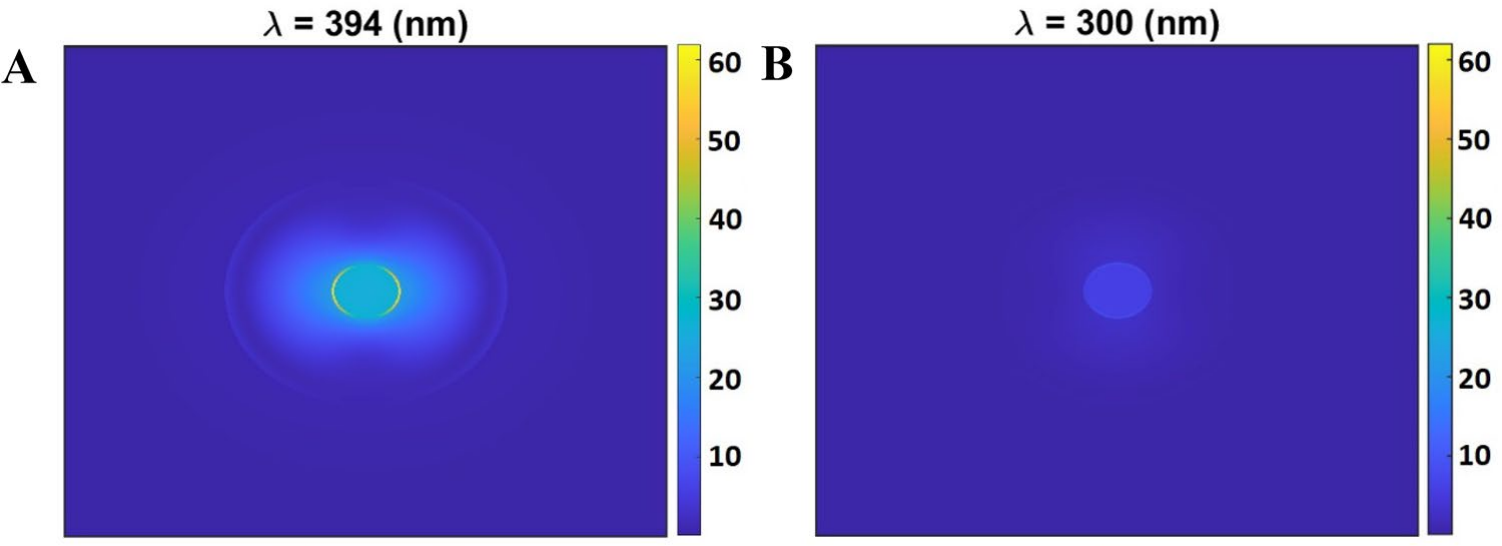
The calculated 2D Electric field (magnitude) distribution of the 30 nm Ag/ZnO nanoparticles at (**A**) Resonance wavelength (394 nm) and (**B**) off-resonance wavelength (300 nm).

Figure 3A and B demonstrate the electric field intensity profile at resonance and off-resonance wavelengths for an Ag/ZnO nanoparticle with a 30 nm diameter. The field enhancement is obvious at LSPR resonance, in which the light is enhanced and confined by the nanoparticle. The field enhancement at the resonance wavelength is 38 times greater than the intensity of the incident field, which shows a strong plasmonic effect. The field distribution and enhancement by the nanoparticle, as an open resonator, increase the local density of states and hence can facilitate the absorption process in the nearby Mg porphyrin molecule. Besides, the scattering plots reveal that introduced nanoparticles with a diameter of 30 nm exhibit a scattering peak at a wavelength of 394 nm. Therefore, it can be claimed that these dimensions are optimal for enhancing the absorption of the chlorophyll molecule.

#### Plasmonic enhancement of the photosynthesis process

**Figure 4:**
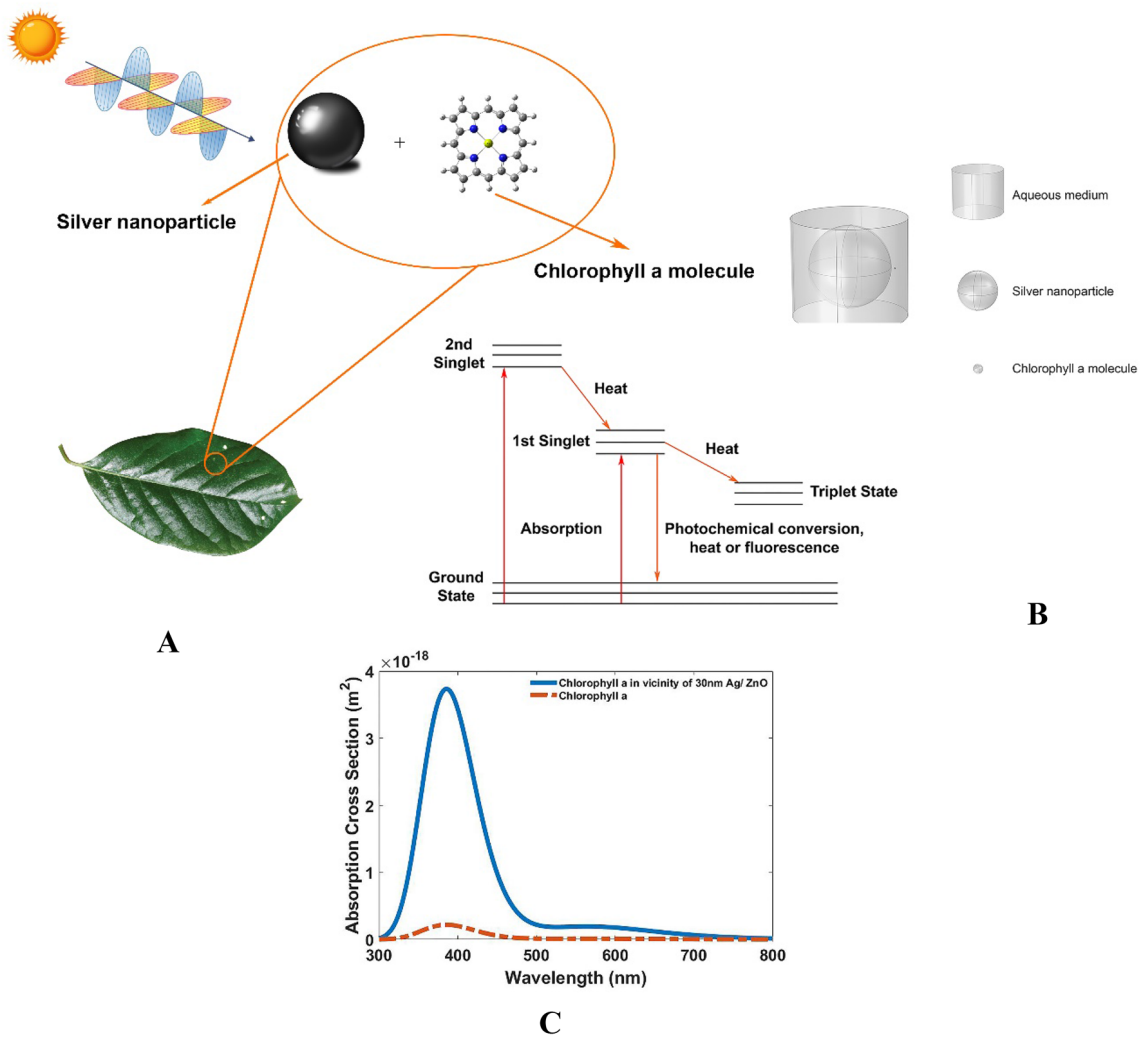
Schematic diagram of the (**A**) presence of Ag/ZnO nanoparticles with a diameter of 30 nm in the vicinity of the porphyrin molecule within the plant leaves exposed to sunlight. The inset depicts the energy levels of the pigment molecule. (**B**) FEM simulation Set-up of 30 nm Ag/ZnO nanoparticles in the water medium in the spectral range of 300 to 800 nm. (**C**) Absorption cross-section of Chlorophyll-*a* molecule in the presence (dashed line) and absence (solid line) of Ag/ZnO nanoparticle. The increase in absorption due to the plasmonic effect is visible.

Figure 4 illustrates a schematic drawing of 30 nm Ag/ZnO nanoparticles in the vicinity of the porphyrin molecule within the plant leaf. The inset shows a graph of the energy levels of a chlorophyll molecule. By absorbing red and blue lights, the excited molecule experiences a transition from the ground to the first or second single excited states, respectively. The emission of energy in the form of heat induces the transition from the second excited state to the first excited state. In the first excited state, photochemical conversion takes place, which competes with energy loss in the form of heat or fluorescence emission. Sometimes a long-lived triplet excited state occurs, leading to the production of harmful single oxygen. To prevent this, it is necessary to de-excitation of the triplet state^60^. Nanomaterials Conjugated with light-harvesting molecules absorb a wider spectrum of wavelengths. Conjugated Nanoparticles with the photosynthetic system considerably improve the generation rate of excited electrons due to the effect of plasmonic enhancement. These excited electrons can be utilized for chemical reactions and as a result, the efficiency of the photosystem increases^61^.

Figure 4B shows the simulation region of chlorophyll* a* molecule in an aqueous medium in the vicinity of a Ag/ZnO nanoparticle with a diameter of 30 nm. The absorption rate of the chlorophyll molecule in the presence and absence of Ag/ZnO nanoparticles is shown in Fig. 4C. The enhancement is obvious when the Ag/ZnO nanoparticle was added to the medium, which is mainly due to the plasmonic effects that increase the generation rate of excited electrons. These excited electrons cause more chemical reactions and enhancement of chemical reactions^61^. The data reported in this study reveal that the behavior of chlorophyll* a* molecule has changed in the presence of Ag/ZnO nanoparticles. The increase in the absorption rate of the chlorophyll molecule is evident in Fig. 4C, which occurs owing to the oscillation of Plasmon on the surface of Ag/ZnO nanoparticles. This enhances the rate of photosynthesis which has a significant effect on the fast cultivation of plants. The increase in photosynthetic efficiency via the plasmonic effect is attributed to the SPRs of Ag/ZnO nanoparticles, which enhances the light-harvesting by pigments and increases the production of chemical energy by the plant. A 16,368% increase in the absorption rate of the chlorophyll molecule is observed in the presence of Ag/ZnO nanoparticles compared to the absence of Ag/ZnO nanoparticles.

### Experimental results

#### TEM analysis of the spherical Ag/ZnO composite NPs

**Figure 5:**
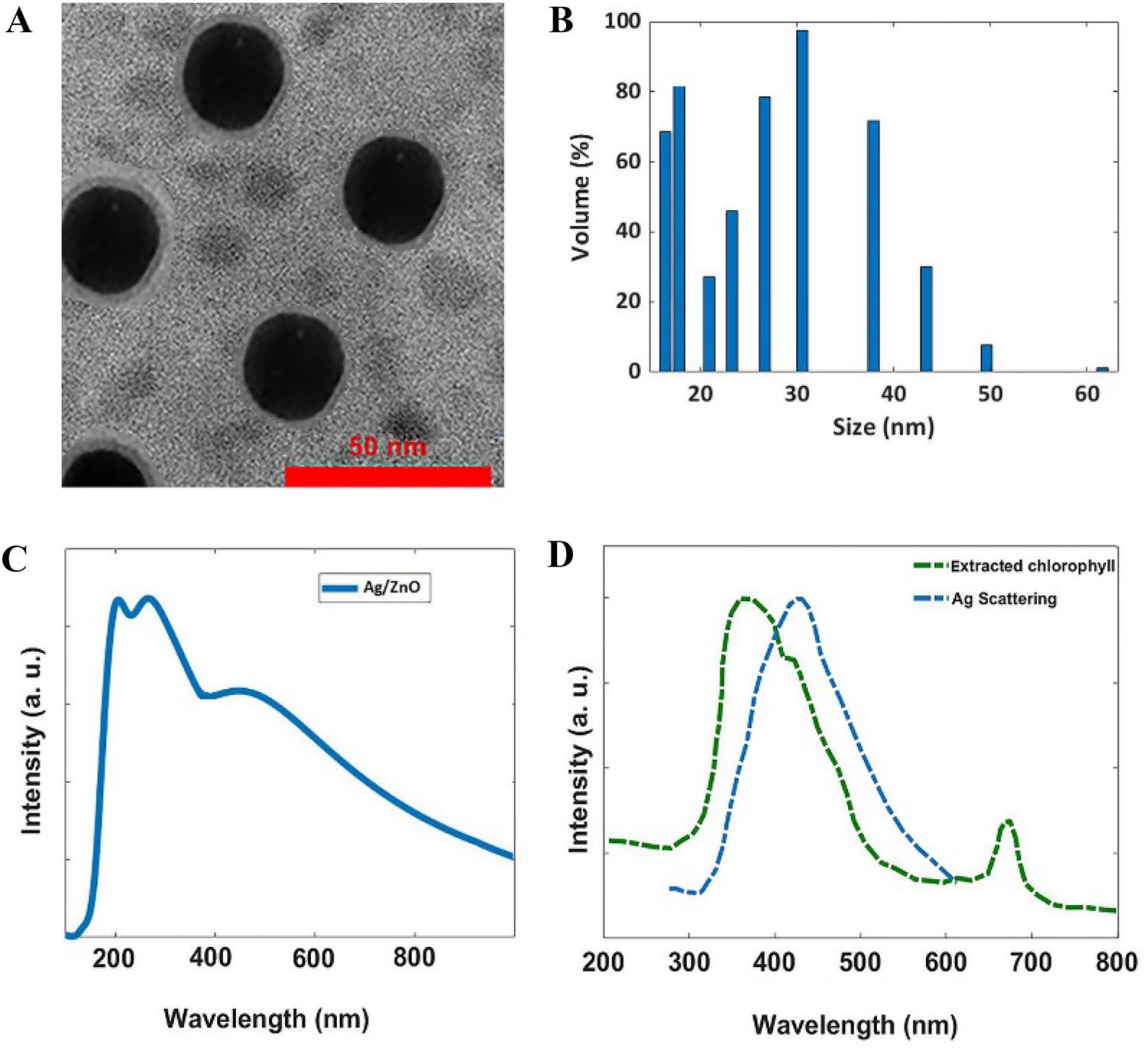
The physicochemical properties of synthesized Ag/ZnO composite nanoparticles. (**A**) TEM Image of the synthesized Ag/ZnO nanoparticles; (**B**) Distribution of the synthesized nanoparticles; (**C**) UV–Vis spectrum of the synthesized nano-colloid and, (**D**) overlapping between Ag scattering spectrum and UV–Vis spectrum of the extracted chlorophyll.

The TEM microscopy analysis result shows there are two types of nanoparticles in the image. Ag/ZnO nanoparticles are 30 nm and there are nanoparticles beside the Ag/ZnO nanoparticles with a diameter of 12.5 nm (Fig. 5A). Similar results have been reported in the literature^62,63^.

#### DLS and zeta-potential analysis

Information from DLS measurement indicates the most dispersion of particles is about 12-15 nm and 30 nm with relatively homogeneous distribution as confirmed by TEM results (Fig. 5B). Surface charge potential (from zeta potential) values of synthesized nanoparticles show + 8.9 mV. It was stated that produced nanoparticles had positively charged on their surface. When the particles dispersed in a solvent have a large negative or positive zeta potential, they tend to repel each other and have no tendency to agglomerate. But if the zeta potential is low, then there will be no force to prevent the particles from agglomerating^64^. In general, the zeta potential in the range of − 30 mV to + 30 mV shows the optimum potential stability of the metal nanoparticles and prevents NPs from agglomeration in a colloidal solution^62–65^.

#### UV–visible and photoluminescence spectroscopy analysis

The UV absorption spectrum of the synthesized Ag/ZnO nanoparticles was shown in Fig. 5C. The results showed the maximum absorption peaks of 200, 250, and 450 nm. However, the bands are relatively broad and it covers all ranges of the UV-B and UV-A spectra. In agreement with our result, the Ag/ZnO NPs show a UV absorbance at a peak of 404 nm due to its probable Surface Plasmon Resonance (SPR) properties as described by Senthilkumar et al.^65^ and Zamiri et al.^66^. Figure 5D indicates overlapping between absorption of extracted chlorophyll* a* and scattering of Ag nanoparticles and as the figure shows there are good overlapping between these two spectra. It means absorbed wavelengths at 200–400 nm Ag NPs can scatter light with wavelengths between 400 and 500 nm and it can be absorbed by plant’s chlorophyll. It should be mentioned that the extracted chlorophyll is related to chlorophyll* a* and *b*.

### Effect of Ag/ZnO nanoparticles on wheat seed germination rate: impact of concentrations and priming duration

The influence of Ag/ZnO nanoparticles (NPs) on wheat seed germination rate was explored across different NPs concentrations and priming durations. This investigation builds upon the findings of Elizabeth and Rai-Kalal^67,68^, demonstrating the pivotal role of NPs concentrations and treatment durations in affecting germination percentages. Notably, Elizabeth’s work also highlighted the correlation between NPs concentrations and seed priming times in various plant species.

Our study corroborates these findings, as depicted in Fig. S4A, which presents the outcomes of seed priming with Ag/ZnO NPs at varying concentrations and durations. Particularly striking is the observation that priming wheat seeds with Ag/ZnO NPs at 15 mg/L for 12 h achieved an impressive germination rate of 99.33%. This rate represents an 18% increase over the control group. A parallel study by Rai-Kalal and Jajoo revealed a comparable outcome, reporting a 14% elevation in germination rate in wheat seeds treated with ZnO NPs at 10 mg/L for 18 h. The essential role of Zn ions in seed germination processes is well-documented^69,70^, influencing factors such as water absorption, ABA hydrolysis, gibberellic acid (GA3) biosynthesis, and root and shoot priming. Yet, the role of Ag NPs on germination remains less definitive. While Vannini et al.^71^ found no alteration in germination rate in wheat seeds treated with Ag NPs at 10 mg/L, López-Luna et al.^72^ reported similar findings for Ag@CoFe_2_O_4_ NPs priming. Notably, our study unraveled that exceeding 15 mg/L Ag/ZnO NPs concentrations and 12 h of priming led to a substantial decline of 43–75% in germination rates.

### Morphological parameters and growth responses

Beyond germination rate, our investigation delved into various morphological parameters, including shoot and root length, as well as fresh and dry weights of propagated seeds. These parameters were assessed to discern the impact of NPs concentration and priming time on seedling growth. A notable trend emerged from our findings, revealing that varying NPs concentrations during different priming durations significantly influenced the radical and plumule length of wheat seedlings. The interplay between NPs concentration and priming duration also manifested in the growth rate of seedlings. For instance, while low Ag/ZnO NPs concentrations (up to 5 mg/L) correlated with reduced shoot growth, higher concentrations exhibited a progressive increase, peaking at 15 mg/L. Similarly, under distinct priming times of 18 and 24 h, seedlings displayed a gradual rise in shoot length followed by a decline at higher NPs concentrations. Remarkably, the control group subjected to 12 h of priming without Ag/ZnO NPs showed the highest shoot length. Root growth exhibited a parallel trend, showcasing the pivotal role of Zn ions in early coleoptile and radicle development. Further support for this was found in the study by Broadley et al.^73^, which underscored Zn’s function as a co-factor in enzymatic processes involved in carbohydrate metabolism and protein synchronization during seed germination. Rai-Kalal and Jajoo^68^ suggested that the nanometric size and assimilation capacity of ZnO NPs contributed to enhanced shoot and root growth, and our findings echoed this relationship. Interestingly, our study found that the release and absorption of Zn^2+^ were influenced by Ag^+^ shell or gradual Zn^2+^ release from Ag/ZnO NPs. This interaction aligned with the observed growth patterns in our study, demonstrating the intricate interplay between NPs and growth responses. It's noteworthy that Ag^+^ ions appeared to impede water uptake and cell division processes, impacting seedling fresh and dry weights, which were closely tied to germination rates and shoot/root lengths.

### Seedling vigor and implications

*While all priming treatments led to decreased seedling vigor and vigor indices compared to the control, seedling vigor* index-I exhibited a notable increase in seedlings hydro-primed for 12 h. This phenomenon aligns with the superior germination rates and growth characteristics observed in these seedlings. Rai-Kalal and Jajoo^68,74^ reported similar high vigor indices in ZnO NP-primed seeds. In light of these outcomes, seedlings hydro-primed for 12 h emerged as a promising choice for further experiments, as they exhibited enhanced germination rates, growth characteristics, and vigor indices. This selection paves the way for more targeted investigations into the intricate relationships between Ag/ZnO NPs, priming durations, and seedling development.

### Impact of light conditions and Ag/ZnO NPs concentrations on wheat morphophysiological properties

In the realm of plant growth and development, the manipulation of red and blue lights within LED-based artificial lighting systems has emerged as a potent regulator. These lighting systems, celebrated for their cost-effectiveness, extended operational lifetimes, and precise wavelength specificity, offer a promising avenue for enhancing crop outcomes^75^. Particularly noteworthy is the capability of red light LEDs to facilitate wheat maturity, while augmenting plant growth rate and seed yield through the integration of blue LEDs^76^. The studies conducted by Cope and Bugbee^77^ and Dougher and Bugbee^78^ unveiled the wheat’s subdued sensitivity to blue light dosage induction.

**Figure 6:**
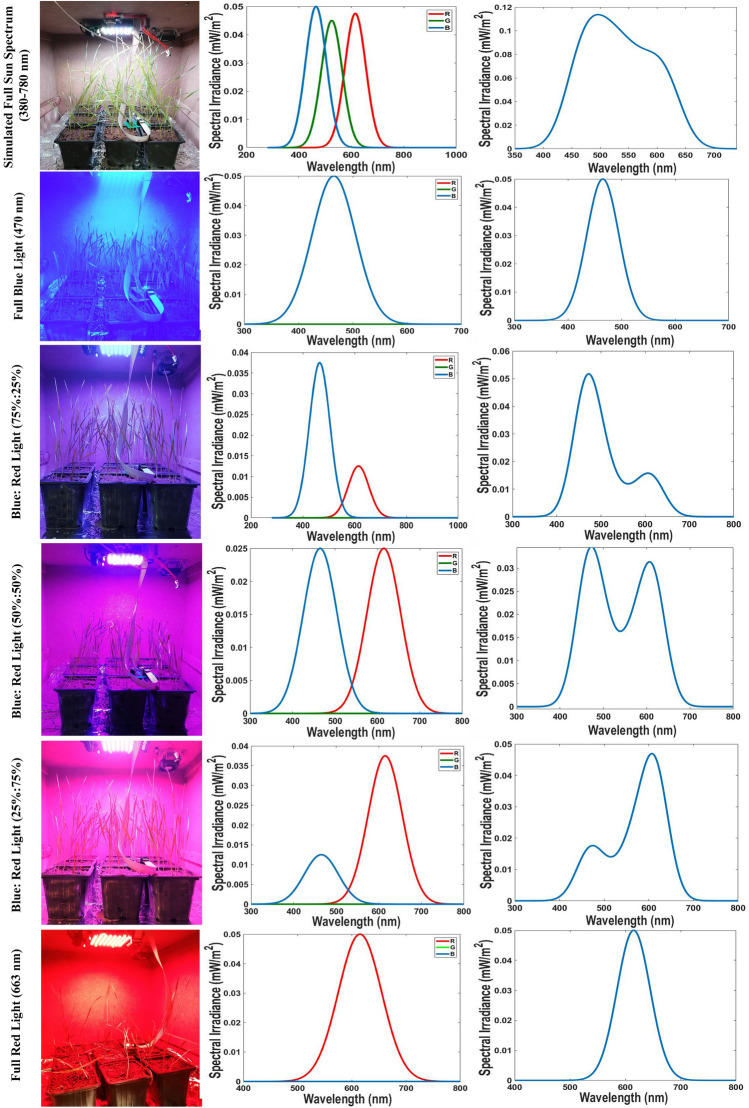
The simulated six different spectrums in an LED-supplemented benchtop speed breeder box. The spectrum irradiance (W/m^2^) of the RGB sensor and LED panel spectrums were plotted by MATLAB software in the range of 380–780 nm.

Intriguing insights from Vikas et al.^79^ illuminated the potential of red LED light in steering wheat cultivars through five generations annually. Under carefully curated conditions, these cultivars displayed a four-leaves phase within 36–42 days and physiological maturity within 67–73 days. Such accelerated growth patterns were orchestrated under artificial red LED light and dark cycles within a temperature range of 17–22 °C. Delving further into the intricate realm of plant-NPs interaction, the penetration of nanoparticles (NPs) through roots and shoots has been recognized, engendering multifarious modifications in morphological and physiological attributes of plant species at cellular and subcellular levels^80–82^. The transformative impacts of NPs hinge upon factors encompassing application methods, physicochemical attributes, and concentration levels. Soil application, foliar spray, and seed treatment have emerged as common conduits for plant growth analysis under diverse NP types and concentrations. Experimental contexts encompassing plate growth mediums, hydrophobic setups, and pot conditions have unveiled the potential for promoting seed germination^82^ and hastening plant growth and development^83–85^, provided NPs are applied at concentrations below toxic thresholds. To probe these dynamics further, hydro-primed seedlings, exhibiting optimal growth rates following 12 h of priming, were transplanted into polyethylene pots (10 cm × 10 cm × 15 cm) harboring greenhouse soil. These pots were subsequently introduced into an adjustable speed breeding chamber, allowing meticulous control over light conditions, intensity, temperature, and photoperiod. Among the seven distinct light conditions meticulously selected were natural sunlight (field condition, 4500 lx), simulated full sun spectrum (4500 lx), blue light (470 nm) at 100%, B75%: R25%, B50%: R50%, B25%: R75%, and red light (663 nm) at 100%. Natural sunlight was designated as the control light condition, providing a baseline for comparative analysis. Detailed RGB sensor data and LED panel spectra for all light conditions can be found in Fig. 6. The ensuing investigation pivoted towards the morphophysiological aspects of wheat plants subjected to varying concentrations of Ag/ZnO NPs (ranging from 0 to 50 mg/L) within each light condition. This comprehensive analysis, spanning across the 30-day period post-planting, unfolded within an intricately adjusted speed breeding chamber operating under a 16-h light/8-h dark photoperiod at a constant 24 °C. Notably, this exploration delved into uncharted territory, charting new frontiers in understanding the interplay between Ag/ZnO NPs concentrations, light conditions, and wheat's morphophysiological responses.

#### Shoot morphological properties of wheat plants

Figure S10 portrays the comparative mean analysis of shoot lengths in various treatment conditions. When excluding Ag/ZnO NPs colloids, an upsurge in the intensity of red wavelength (663 nm) was observed to induce a gradual augmentation in the shoot length of wheat plants. Notably, the most remarkable shoot length was observed in wheat plants exposed to a full 663 nm red-light regimen, boasting an average length of 125.40 mm. Impressively, this measurement stood 1.7-fold and 1.3-fold higher than their counterparts under natural sunlight and simulated sunlight conditions, respectively. Moreover, wheat plants nurtured in simulated sunlight exhibited a 30% growth increase over their natural light-exposed counterparts. However, a stark decline in shoot length, amounting to 62%, was observed under full blue light conditions compared to natural sunlight. Remarkably, the inclusion of Ag/ZnO NPs at concentrations up to 15 mg/L exhibited a significant shoot length enhancement, showcasing its pronounced influence on wheat plant shoot elongation. Paradoxically, higher NP concentrations appeared to hinder shoot length growth in plants subjected to sunlight and red-blue light conditions. Notably, the pinnacle of shoot length, measuring 127.89 mm, was achieved in plants cultivated under full red light (663 nm) conditions and treated with 50 mg/L Ag/ZnO NPs colloid (Fig. S10). However, the scenario for shoot diameter was different, as plants nurtured under red and blue light conditions displayed a marked reduction compared to the control plants basking in natural sunlight. Strikingly, the most expansive shoot diameter, averaging 2.15 mm, was recorded in plants thriving under simulated full sun spectrum conditions and treated with a 15 mg/L Ag/ZnO NPs suspension. This measurement triumphed as 1.36-fold larger than that of control plants cultivated under natural sunlight. Although heightened red light appeared to incrementally enhance wheat plant shoot diameter, the growth rate in diameter dwindled in the presence of higher concentrations of Ag/ZnO NPs (Fig. S11). Further investigation encompassed the determination of leaf count per plant across various treatments. Intriguingly, no discernible variance emerged in leaf number between plants nurtured under natural and simulated sunlight conditions, regardless of the concentration of Ag/ZnO NPs. However, elevating blue light intensity instigated a consequential reduction in plant growth rate, consequently leading to a decrement in leaf count (Fig. S12). The outcomes resonate with the findings of Dong et al.^86^, which delineate how red light favors wheat seedling height over control plants, while blue light at the seedling stage suppresses growth, culminating in dwarfism. This intricate balance might be rooted in the potential inverse relationship between red light and the POD enzyme, which potentially induces growth and stem extension through POD deactivation under full red light conditions^87^. In the tapestry of light combinations, a gradual augmentation in red light correlated with a noteworthy increase in shoot fresh weight (SFW). However, the introduction of NPs led to a significant attenuation in weight gain, precipitating a noticeable decline in SFW in plants treated with 50 mg/L NPs compared to the control plants. Further scrutiny of shoot weight measurements indicated that the highest SFW occurred in plants nurtured under simulated sun spectrum conditions and treated with Ag/ZnO NPs at a concentration of 5 mg/L, exhibiting a remarkable 1.87-fold increase compared to natural sunlight (Fig. S13). Conversely, a substantial reduction manifested in the shoot dry weight (SDW) of plants under red-light conditions, further exacerbated by NP treatment. The SDW findings pointed towards the likelihood that heightened plant height in red light conditions could be attributed to water uptake and shoot elongation, rather than cell division and biomass production. Intriguingly, the SDW in plants exposed to simulated sun spectrum conditions and treated with a 15 mg/L Ag/ZnO NPs suspension stood 25.5% higher than that of those under natural sunlight (Fig. S14). A comprehensive examination of the SDW to SFW ratio elucidated that the most elevated value was associated with plants thriving under a blue 75%: red 25% light composition, concurrently treated with 50 mg/L Ag/ZnO NPs (Fig. S15). This intriguing outcome mirrors the positive influence of Zn ions on the biosynthesis of natural auxin (IAA), thereby triggering cell division activation and enlargement, ultimately culminating in elevated biomass production. This phenomenon finds consonance with prior findings by Ali et al.^88^.

#### Root morphological properties of the wheat plants

**Figure 7:**
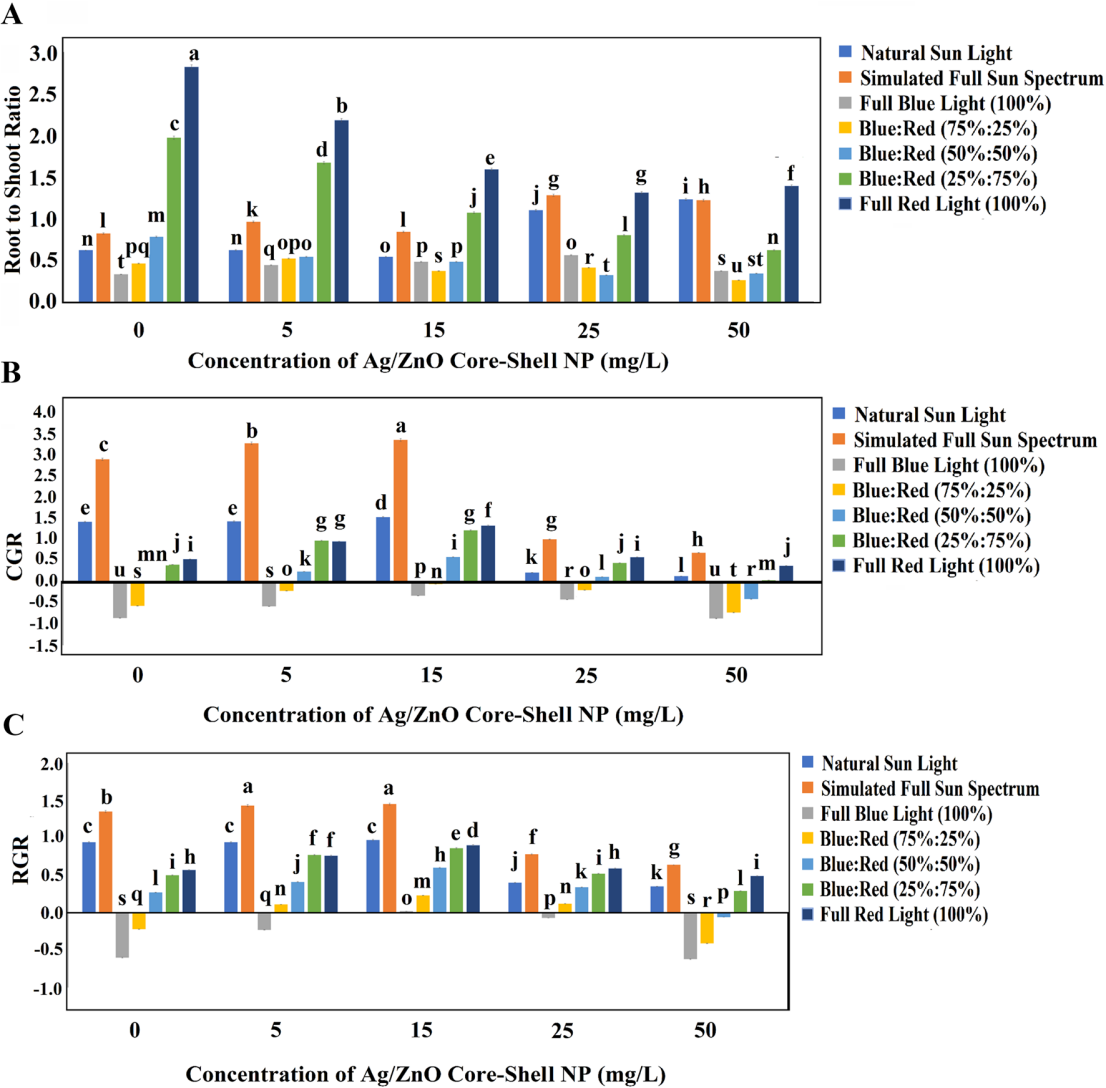
The means of growth parameters in 1-month-old wheat plants in different light conditions and Ag/ZnO NPs concentrations. (**A**) The root-to-shoot biomass ratio; (**B**) The crop growth rate (CGR); (**C**) The relative growth rate (RGR). The bars represent SD.

Root growth and shoot growth exhibited a robust interplay in NPs untreated control plants, revealing a pronounced correlation with light conditions. Notably, the incremental rise in red light (663 nm) engendered a noteworthy reduction in root length. This manifested as a 52.07% and 63.02% decrease under full red-light conditions when juxtaposed with full blue light and natural sunlight conditions, respectively. In the presence of Ag/ZnO NPs, the curtailment of root length was accentuated, particularly in wheat plants subjected to blue-red light amalgamation. Remarkably, when compared to NPs-untreated control plants exposed to natural sunlight, a remarkable 1.3-fold augmentation was observed in root length among plants cultivated under simulated sunlight. A pinnacle in root length emerged when plants were treated with 15 mg/L Ag/ZnO NPs under simulated sunlight conditions, showcasing a striking 1.53-fold increase compared to NPs-untreated control plants under natural sunlight conditions. However, as NP concentration ascended, root length experienced significant diminution (Fig. S16). Akin trends manifested in root diameter and root number attributes, wherein augmented red light intensity and NP concentration resulted in notable root diameter reduction. The zenith root diameter, averaging 0.81 mm, was documented in plants thriving under simulated full sun spectrum conditions, concurrently treated with a 5 mg/L Ag/ZnO NPs suspension (Fig. S17). Root number assessment unveiled that heightened red-light intensity and restrained Ag/ZnO NPs concentration contributed to substantial root number amplification in plants. Consequently, the zenith root number per plant, averaging 6.4, was observed among plants cultivated under the simulated sun spectrum in tandem with 15 mg/L or 50 mg/L Ag/ZnO NPs suspension (Fig. S18). In the context of root morphological properties, the maximum fresh weight of roots was noted in plants under the simulated sun spectrum, devoid of Ag/ZnO NPs treatment, triumphing as nearly twofold superior to natural sunlight conditions (Fig. S19). Interestingly, no significant difference emerged between the root fresh weights (RFWs) of NPs-untreated plants and those treated with 15 mg/L Ag/ZnO NPs under simulated sunlight conditions. Conversely, concerning root dry weight, an increment of 34% was observed in NP-untreated plants through an intensified red light, whereas a 55% escalation was witnessed in root dry weight among plants treated with 15 mg/L Ag/ZnO NPs under full red-light conditions. Intriguingly, the apex root dry weight, surpassing all others, was attained in plants treated with 15 mg/L Ag/ZnO NPs under simulated sun spectrum conditions, towering as a staggering 2.5-fold increase over the control plant (Fig. S20). Delving into ratios, the analysis of root dry weight (RDW) to root fresh weight (RFW) ratio underscored the preeminence of plants treated with 15 mg/L Ag/ZnO NPs (Fig. S21). However, noteworthy alterations in light conditions didn't mirror a commensurate shift in this parameter. Conversely, compared with the control plant, no substantial divergence manifested in the root-shoot biomass ratio among wheat plants treated with 15 mg/L Ag/ZnO NPs under the simulated sun spectrum and their NPs-untreated counterparts under natural sunlight conditions. Nevertheless, an escalation in Ag/ZnO NPs concentration and red-light intensity elicited a notable boost in the root-shoot biomass ratio (Fig. 7A).

#### Ag/ZnO NPs and simulated solar spectrum enhance wheat growth rate

Gauging the vital parameters for growth rate determination in crops, namely the relative growth rate (RGR) and the cumulative growth rate (CGR), illuminated significant transformations with escalating Ag/ZnO NPs concentration, up to 15 mg/L. Remarkably, the zenith values emerged among plants basking in simulated sunlight in tandem with a 15 mg/L Ag/ZnO NPs presence (Fig. 7B and C). This configuration heralded a maximal growth rate, surpassing the control plant under natural sunlight conditions by a remarkable 2.4-fold enhancement.

#### Chlorophyll *a* and carotenoids content

**Figure 8:**
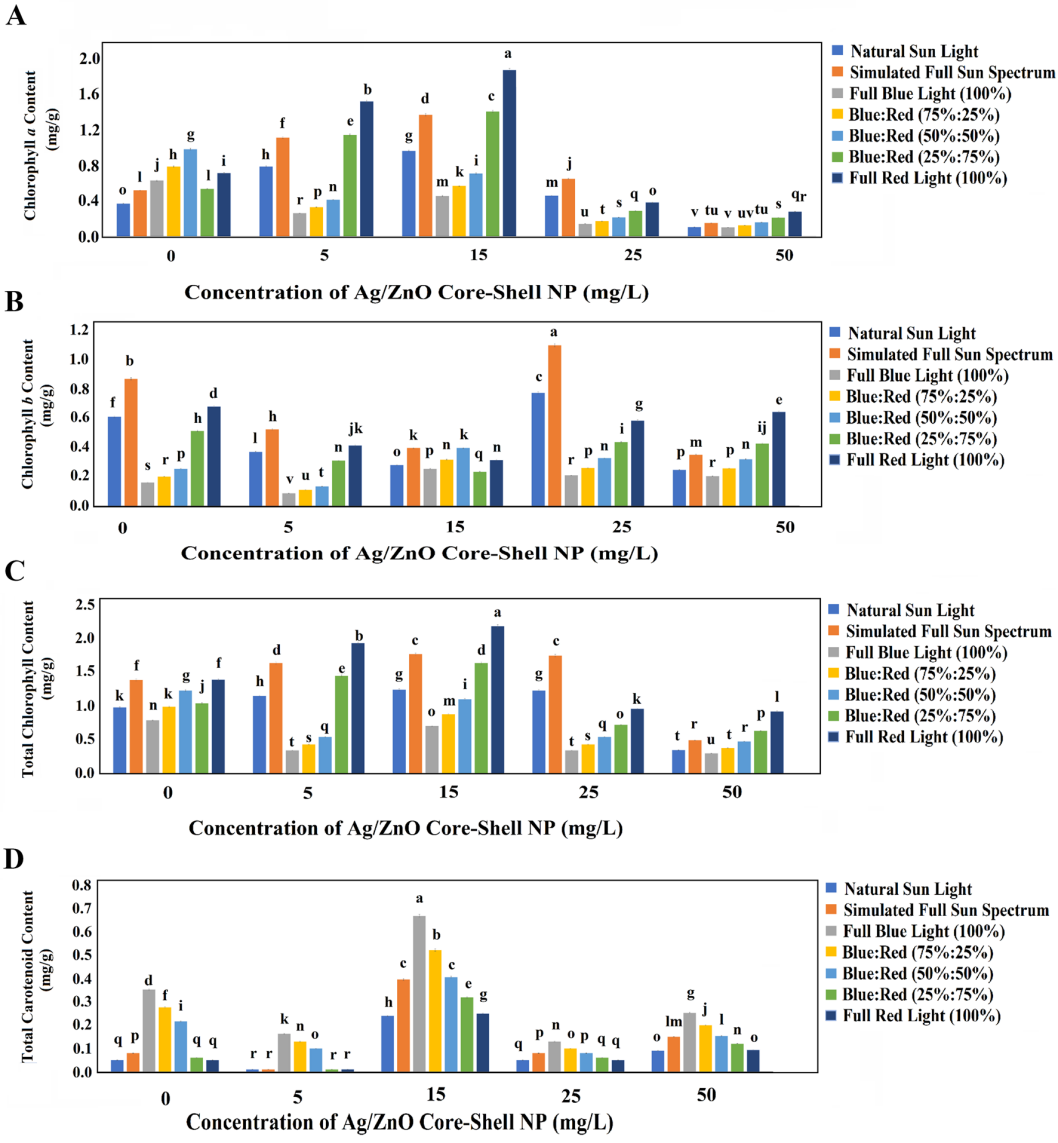
The means of chlorophyll-*a* and carotenoid contents in 1-month-old wheat plants in different light conditions and Ag/ZnO NPs concentrations. (**A**) The means of chlorophyll* a* content (mg/g); (**B**) The means of chlorophyll *b* content (mg/g); (**C**) The means of total chlorophyll content (mg/g); (**D**) The means of carotenoids content (mg/g). The bars represent SD.

The pivotal role of chlorophyll content in reflecting photosynthetic rates and gauging plant health is well-established within the scientific discourse^89^. Consequently, the intricate interplay of light conditions and Ag/ZnO NPs concentration exerted a profound influence on chlorophyll-*a*, chlorophyll-*b*, and total chlorophyll contents. Among NP-untreated control plants, reared under natural sunlight, the chlorophyll-*a* content registered a minimal mean of 0.37 mg/g. Evident shifts in light conditions yielded substantial increments in chlorophyll-*a* content. Notably, under the simulated sun spectrum, the chlorophyll-*a* content scaled to a remarkable 1.4-fold superiority compared to control plants under natural sunlight conditions. An incremental elevation in red light intensity, up to 50%, engendered a gradual surge in chlorophyll-*a* content, accentuating the affirmative impact of blue-red light synergy on bolstering chlorophyll-*a* biosynthesis in wheat. Introducing Ag/ZnO NPs further amplified this phenomenon, culminating in the maximal chlorophyll-*a* content observed in plants treated with 15 mg/L Ag/ZnO NPs, averaging 0.96 mg/g. This value marked a remarkable 2.6-fold augmentation over control plants under natural sunlight conditions. This underscores a robust correlation binding chlorophyll-*a* content, red light conditions, and Ag/ZnO NPs concentration. The apical chlorophyll-*a* content, quantifying 1.87 mg/g, manifested among plants cultivated under full red light (663 nm) and supplemented with 15 mg/L Ag/ZnO NPs, signaling a striking 5.05-fold increase compared to NPs-untreated control plants under natural sunlight conditions (Fig. 8A). Similar dynamics permeated chlorophyll *b* content, evidenced by a notable 42.6% increment in plants thriving under the simulated sun spectrum in comparison to control plants under natural sunlight. In blue-red light conditions, augmented red light intensity elicited a discernible upsurge in chlorophyll *b*, mirroring the trend seen in control plants under natural sunlight conditions. The introduction of Ag/ZnO NPs, accompanied by escalating concentrations, culminated in the zenith chlorophyll *b* content, averaging 1.10 mg/g, observed among plants cultivated under the simulated sun spectrum, supplemented with a 25 mg/L Ag/ZnO NPs suspension (Fig. 8B). The comprehensive evaluation of total chlorophyll content accentuated its maximal augmentation, quantifying a robust 2.22-fold surge among plants treated with 15 mg/L Ag/ZnO NPs concentration under full red light (663 nm) conditions (Fig. 8C). Turning to the carotenoid content, it emerged that the pinnacle of carotenoid content materialized among plants treated with 15 mg/L Ag/ZnO NPs concentration, flourishing under the full blue light (470 nm) condition (Fig. 8D). Conversely, no appreciable mean distinction surfaced between control plants and those nurtured under red light conditions. Notably, the presence of Ag/ZnO NPs elicited a concentration-dependent elevation in carotenoid contents up to 15 mg/L, across all light conditions. Prior research underscores the pivotal role of Ag and ZnO NPs in expediting leaf expansion, curtailing leaf senescence and thylakoid lamellae damage, thereby fostering advanced chlorophyll-*a* and *b* biosynthesis processes in plants^90^. Ehsan et al.^91^ reported analogous findings, detailing significant upticks of 92% in chlorophyll-*a*, 71% in chlorophyll-*b*, and 84% in total chlorophyll in wheat when exposed to 100 mg/L urea and 75 mg/L bimetallic Ag/ZnO alloy NPs. In concurrence with our observations, the studies of Sharma et al.^92^ and Sun et al.^93^ ascertain that Ag and ZnO NPs orchestrate gene expressions that encode regulatory enzymes within the photosynthesis process, consequently fostering substantial enhancements in total chlorophyll content.

#### Total phenol and flavonoid content

The assessment of total phenol and flavonoid content is essential in understanding the plants' biochemical responses to different treatments, shedding light on their defense mechanisms and antioxidative activities. Our investigation revealed intriguing trends in the accumulation of these compounds under varying conditions.

Under the simulated sun spectrum, a reduction of approximately 25% in total phenol content was observed compared to control plants subjected to natural sunlight conditions. This reduction suggests a potential influence of light composition on phenolic compound synthesis. Notably, the plants exposed to full blue light conditions exhibited a remarkable 1.28-fold increase in phenolic content, indicating that blue light may stimulate phenolic biosynthesis. However, as red light intensity increased, a gradual decline in phenolic content was noted, possibly indicating a regulatory role of red light in phenolic accumulation. The examination of total flavonoid content also yielded intriguing outcomes. The impact of light conditions on flavonoid synthesis was evident, with an increase observed under red light conditions and at higher Ag/ZnO NPs concentrations. Specifically, wheat plants treated with 25 mg/L Ag/ZnO NPs under sunlight conditions demonstrated the highest flavonoid content. Interestingly, the presence of red light appeared to counteract this increase in flavonoid content, resulting in a significant decrease in plants treated with 25 mg/L Ag/ZnO NPs. These findings underscore the intricate interplay between light conditions, Ag/ZnO NPs concentrations, and the synthesis of secondary metabolites. While blue light seems to enhance phenolic accumulation, red light may play a dual role in modulating phenolic and flavonoid biosynthesis. Furthermore, the effect of Ag/ZnO NPs on flavonoid content appears to be influenced by both light conditions and NPs concentrations. In summary, the alterations in total phenol and flavonoid content offer valuable insights into the plants' biochemical responses to various treatments. Further investigations are warranted to elucidate the underlying molecular mechanisms and regulatory pathways governing these responses^94^.

#### Estimation of lipid peroxidation

The intricate orchestration between aging, biotic stresses, and the onslaught of abiotic challenges in plants elicits the production of secondary metabolites, such as the pivotal lipid peroxidation product, Malondialdehyde (MDA). This compound stands as a tangible indicator of damage sustained by the crucial two-layer phospholipid plasma membrane enveloping plant cells^95^. Our experimental findings unveil a conspicuous narrative: the Ag/ZnO NPs concentration surging beyond the 15 mg/L threshold culminates in an unprecedented 3.84-fold elevation in MDA content within plants thriving under natural sunlight conditions. As a result, the apogee of MDA content finds its zenith in plants treated with the highest Ag/ZnO NPs concentration of 50 mg/L and reared under unadulterated natural sunlight conditions. Notably, a nuanced dance unfolds within the realm of light conditions; intensifying red light luminance progressively begets an upward trend in MDA content across varying NPs concentrations. It's intriguing to note that the nadir of MDA content surfaced within plants treated with 15 mg/L Ag/ZnO NPs and nurtured under the auspices of full blue light conditions (Fig. S24). Aligned with our observations, a study by 63 postulated a significant reduction in MDA content within nano-primed wheat plants, consequent to the introduction of ZnO NPs. This resonates with the protective and stabilizing role assigned to Zn ions upon biomembranes, insulating them against oxidative assaults and maintaining the impermeability and oxidative equilibrium of plasma membranes^96^.

### Biochemical assays

**Figure 9:**
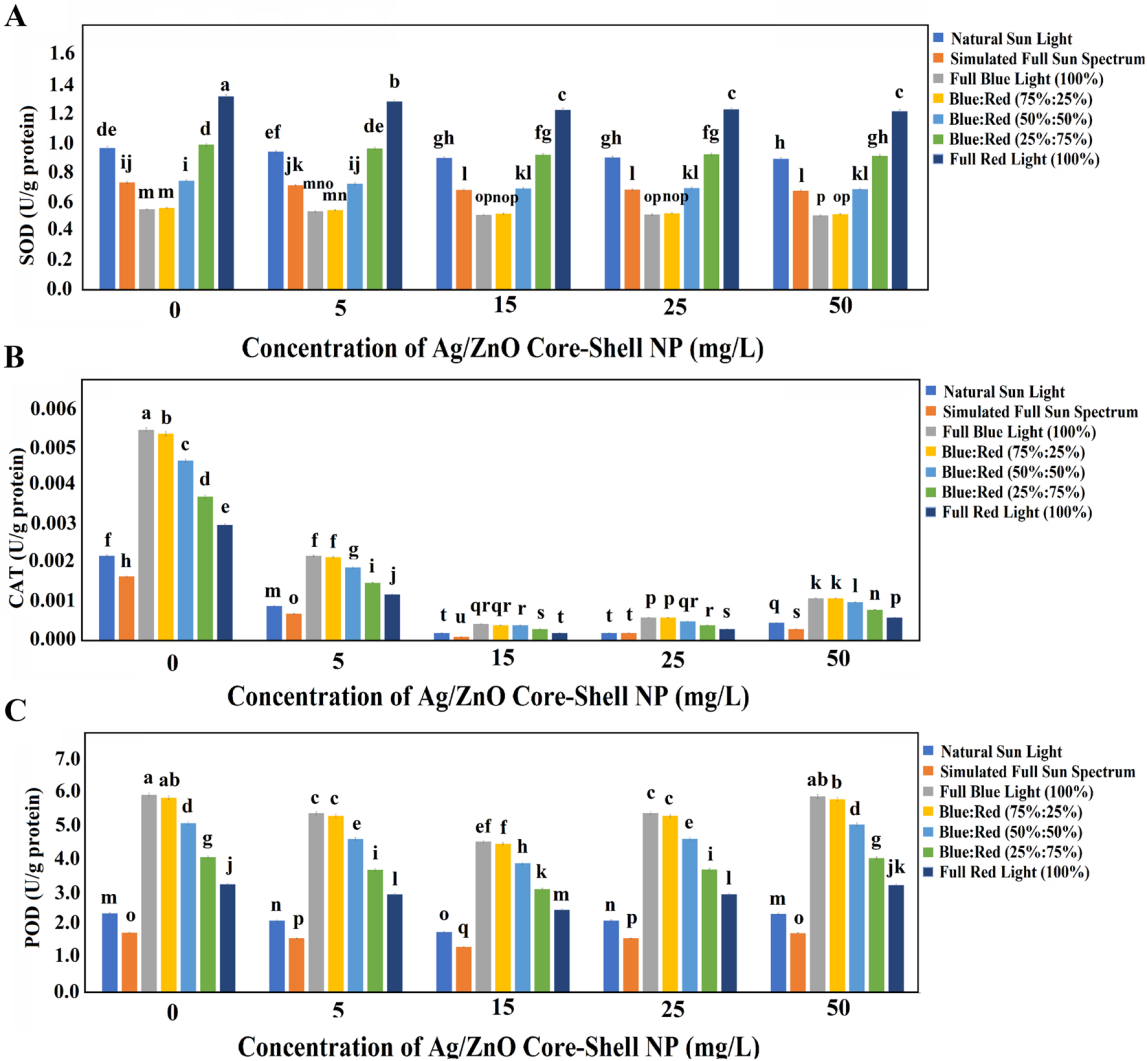
The biochemical assay of enzyme activity in 1-month-old wheat plants in different light conditions and Ag/ZnO NPs concentrations. (**A**) The mean of SOD activity (U/g protein); (**B**) The mean of CAT activity (U/g protein); (**C**) The mean of POD activity (U/g protein). The bars represent SD.

The intricate equilibrium governing the metabolism of reactive oxygen species (ROS) is pivotal in averting oxidative damage within plants. Within the ambit of plant defense mechanisms, a vanguard of antioxidant enzymes – including Peroxidase (POD), superoxide dismutase (SOD), and catalase (CAT) – assumes the mantle of neutralizing prominent free radicals like hydrogen peroxide (H_2_O_2_), superoxide radical (O_2_^−^), and other ROS^68^. Delving into the fascinating realm of antioxidant enzyme activities within wheat plants, subjected to a gamut of treatments, reveals compelling insights. SOD, functioning as the harbinger of O_2_^−^ transformation into H_2_O_2_, exhibited a 28.34% decrement under the simulated sun spectrum conditions compared to the natural sunlight milieu (Fig. 9A). This trend aligns harmoniously with Rai-Kalal and Jajoo's earlier findings^68^. In a symphony of red light augmentation, SOD activity displayed a gradual crescendo. Notably, no perceptible variations in SOD activity emerged across disparate Ag/ZnO NPs concentrations, consistent with findings by Rai-Kalal and Jajoo^68^, who underscored a stark 67% reduction in SOD levels among ZnO NPs-treated plants. Turning our attention to CAT enzymes, diligent protectors of plant cells against oxidative harm and stalwart scavengers of H_2_O_2_, we discerned a crescendo of activity in NP-untreated plants thriving beneath the full blue light conditions – a surge that measured an impressive twofold over the control plants under natural light conditions. As red light intensity burgeoned, a marked decline in CAT activity transpired. This decreasing trend persisted within the milieu of Ag/ZnO NPs concentrations, up to a threshold of 15 mg/L. Significantly diminished CAT activity was unveiled within plants treated with the same NPs concentration under the simulated sun spectrum conditions (Fig. 9B). In contrast, Rai-Kalal and Jajoo’s^68^ findings diverge, pointing to a notable increase in CAT activity among ZnO NPs-primed wheat seedlings. This divergence underscores the potential indirect role of Zn in orchestrating the detoxification of H_2_O_2_ via CAT enzymes, as postulated by Weisany et al.^97^. Lastly, the role of POD enzyme in the scavenging of ROS, thereby mitigating cell oxidative injury, came into focus. A resplendent 2.5-fold surge in POD content materialized within plants thriving under full blue light (470 nm), juxtaposed against NPs-untreated control plants reared in the embrace of natural sunlight. Intriguingly, the ebb and flow of POD content mirrored the rhythm of red light intensity; as the latter surged, the former subsided. Notably, the introduction of Ag/ZnO NPs elicited no significant divergence in POD content across the spectrum of light conditions (Fig. 9C).

## Conclusion

Our findings reveal that the Ag/ZnO composite NPs as an artificial light-harvesting antenna provides an opportunity to overcome the limitations of modern speed breeding programs due to its surface Plasmon resonance effect. According to our findings, the theoretical analysis of the light absorption efficiency of chlorophyll-*a* showed a 160-fold increase in the absorption in the presence of Ag/ ZnO NPs with a 30 nm diameter. We also investigated the impact of chemically synthesized spherical Ag/ZnO composite NPs on the germination rate and its priming efficiency in wheat seeds. The result showed that the seed priming with 15 mg/L Ag/ZnO NPs for 12 h causes an 18% increase in the germination rate compared with hydro-primed wheat seeds. However, the Ag/ZnO NPs have not been recommended for seed priming due to the negative effect of Ag ions on the plant cell division process in plant growth and development. In speed breeding programs, crops are maintained in the adjustable growth chamber associated with light intensity, light wavelength, photoperiod, and temperature conditions to obtain optimized conditions resulting in acceleration of the photosynthesis process and growth rate, which remain challenging. Here, we have evaluated the effect of five different concentrations of Ag/ZnO NPs under natural sunlight conditions as control and six different simulated light condition in the light spectrum simulator system adjusted to 16 h/8 h light/dark photoperiod at 24ºC temperature. The plant morphological analysis results showed an almost 2.5-fold increase in the growth rate of the wheat plants treated with 15 mg/L Ag/ZnO NPs under the simulated full solar spectrum. Our findings related to the physiological analysis showed a 2.6-fold increase in the chlorophyll-*a* content of the plants treated with 15 mg/L Ag/ZnO NPs colloid under the natural sunlight condition, while its content reached 5.058-fold under the full red-light condition. Our findings indicate no obvious oxidative damage and lipid peroxidation of the plasma membrane of plant cells treated with 15 mg/L Ag/ZnO NPs under the simulated solar spectrum. Concerning the consequences, the germination rate, photosynthesis performance, and growth rate of crops are enhanced by the spherical Ag/ZnO composite NPs under the simulated full solar spectrum in the sun simulator device. However, the effect of accumulation of the Ag/ZnO NPs in the plant tissue and its effect on the physiological properties of the treated plants and their interaction with soil microorganisms need to be investigated for further analysis.

### Supplementary Information

Supplementary Figures.

## Data Availability

The datasets used and/or analyzed during the current study are available from the corresponding author upon reasonable request.
