# Supplementary material for: Ag/ZnO core–shell NPs boost photosynthesis and growth rate in wheat seedlings under simulated full sun spectrum
Source: Sci Rep. 2023 Sep 1;13:14385. doi: 10.1038/s41598-023-41575-7 (PMC10474060; doi:10.1038/s41598-023-41575-7)
Supplement: Supplementary file 1 — Supplementary Figures. [file 41598_2023_41575_MOESM1_ESM.docx]

**Ag/ZnO Core-Shell NPs Boost Photosynthesis and Growth Rate in Wheat Seedlings under Simulated Full Sun Spectrum**

**Shahnoush Nayeri^1^, Mahboubeh Dolatyari^1^, Neda Mouladoost^2^, Saeed Nayeri^2^, Armin Zarghami^2^, Hamit Mirtagioglu^3^, and Ali Rostami^1,2*^**

^1^SP-EPT Lab., ASEPE Company, Industrial Park of Advanced Technologies, Tabriz, Iran, [shahnoush.nayeri@hotmail.com](mailto:shahnoush.nayeri@hotmail.com), [m.dolatyari@uni-koeln.de](mailto:m.dolatyari@uni-koeln.de), ORCID ID: <https://orcid.org/0000-0003-0173-0980>, [rostami@tabrizu.ac.ir](mailto:rostami@tabrizu.ac.ir); ORCID ID: <https://orcid.org/0000-0002-8727-4711>

^2^Photonics and Nanocrystal Research Lab. (PNRL), Faculty of Electrical and Computer Engineering, University of Tabriz, Tabriz-51666, Iran, [rostami@tabrizu.ac.ir](mailto:rostami@tabrizu.ac.ir); ORCID ID: <https://orcid.org/0000-0002-8727-4711>, [zarghamiarmin@gmail.com](mailto:zarghamiarmin@gmail.com), [movladoost@ms.tabrizu.ac.ir](mailto:movladoost@ms.tabrizu.ac.ir), Nayeri.s@hotmail.com

^3^Department of Statistics, Faculty of Science and Literature, University of Bitlis Eren, Bitlis, Turkey

***Corresponding author:** Photonics and Nanocrystal Research Lab. (PNRL), Faculty of Electrical and Computer Engineering, University of Tabriz, Tabriz-51666, Iran. E-mail: [rostami@tabrizu.ac.ir](mailto:rostami@tabrizu.ac.ir); ORCID ID: <https://orcid.org/0000-0002-8727-4711>


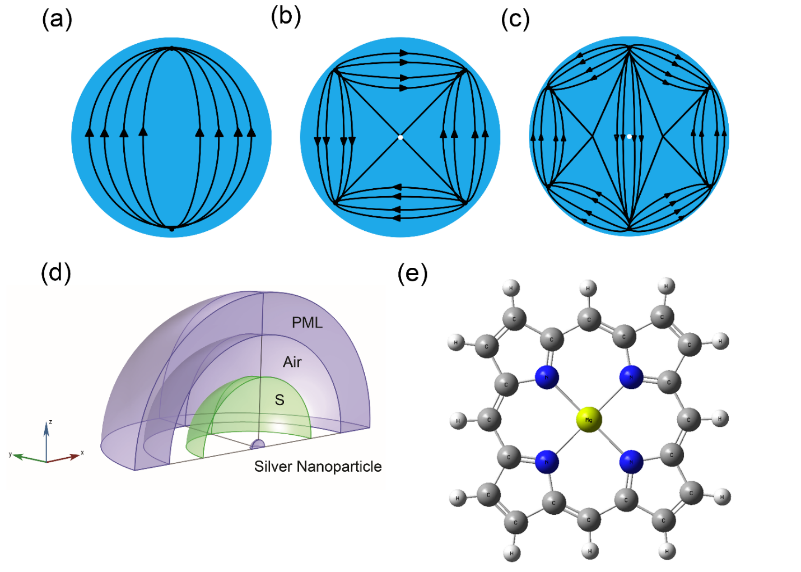


**Figure S1.** Electric field lines of (a) dipole, (b) quadrupole, and (c) octupole for a spherical particle supporting Mie resonances (69), (d) The geometry of spherical silver nanoparticles used in Mie scattering theory. The thickness of PML is considered 250 nm to ensure an acceptable absorption on the boundaries. The inner layer is positioned at a distance of 350 nm from the center of the particle and the outer layer is located at a distance of 600 nm from the center of the particle. The thickness variation from 100 to 500 nm did not have a significant effect on the accuracy of the results, (e) The molecular structure of Mg porphyrin. 20 carbon atoms form the macrocycle, which is shown in gray in the figure. Four nitrogen atoms are placed inside the ring and represented in blue. The complement of the chlorophyll molecule is a magnesium ion (Mg^2+^), which forms a chelate with four nitrogen atoms in the center of the ring.


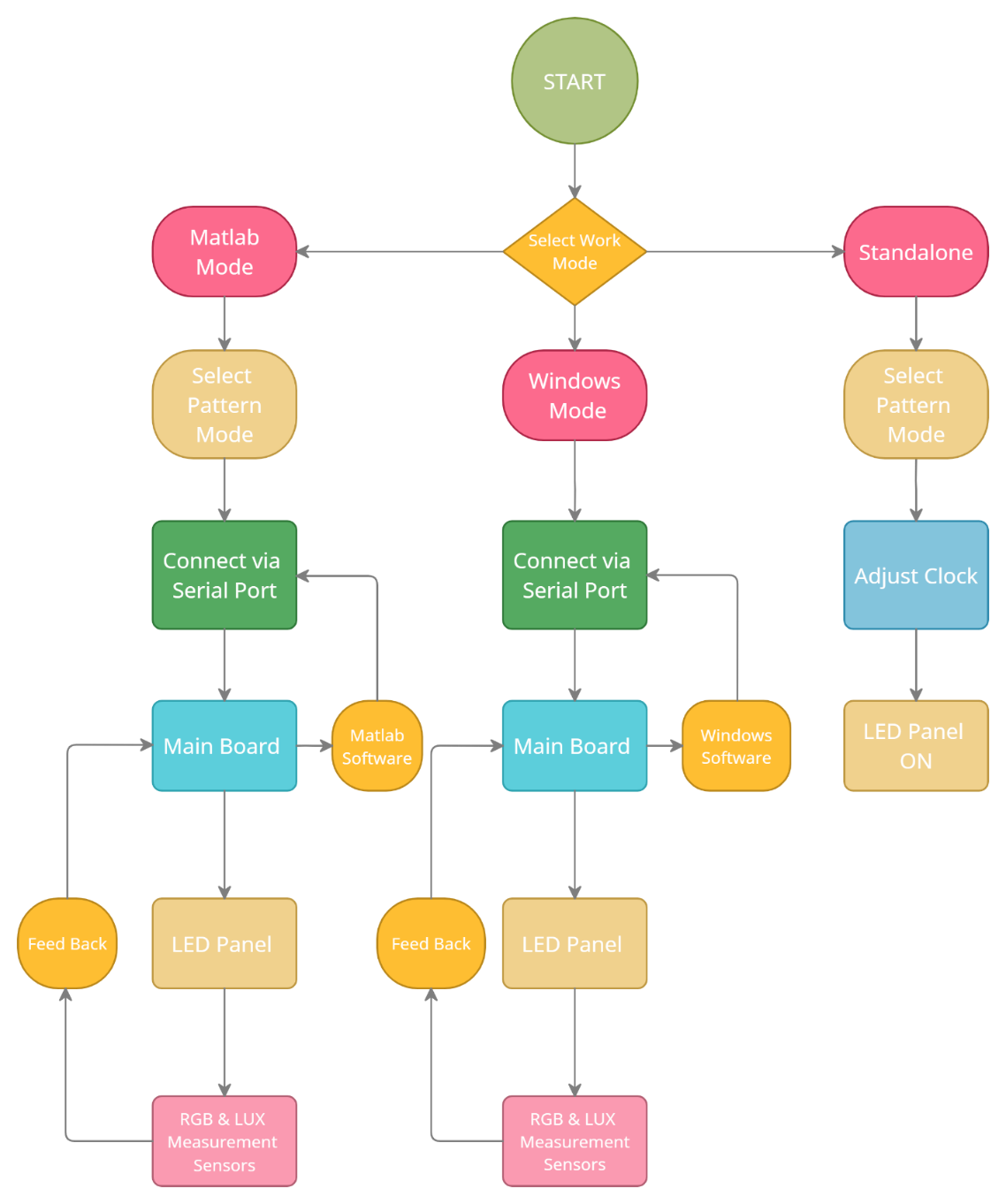


**Figure S2:** Block diagram of the speed breeder device.

**
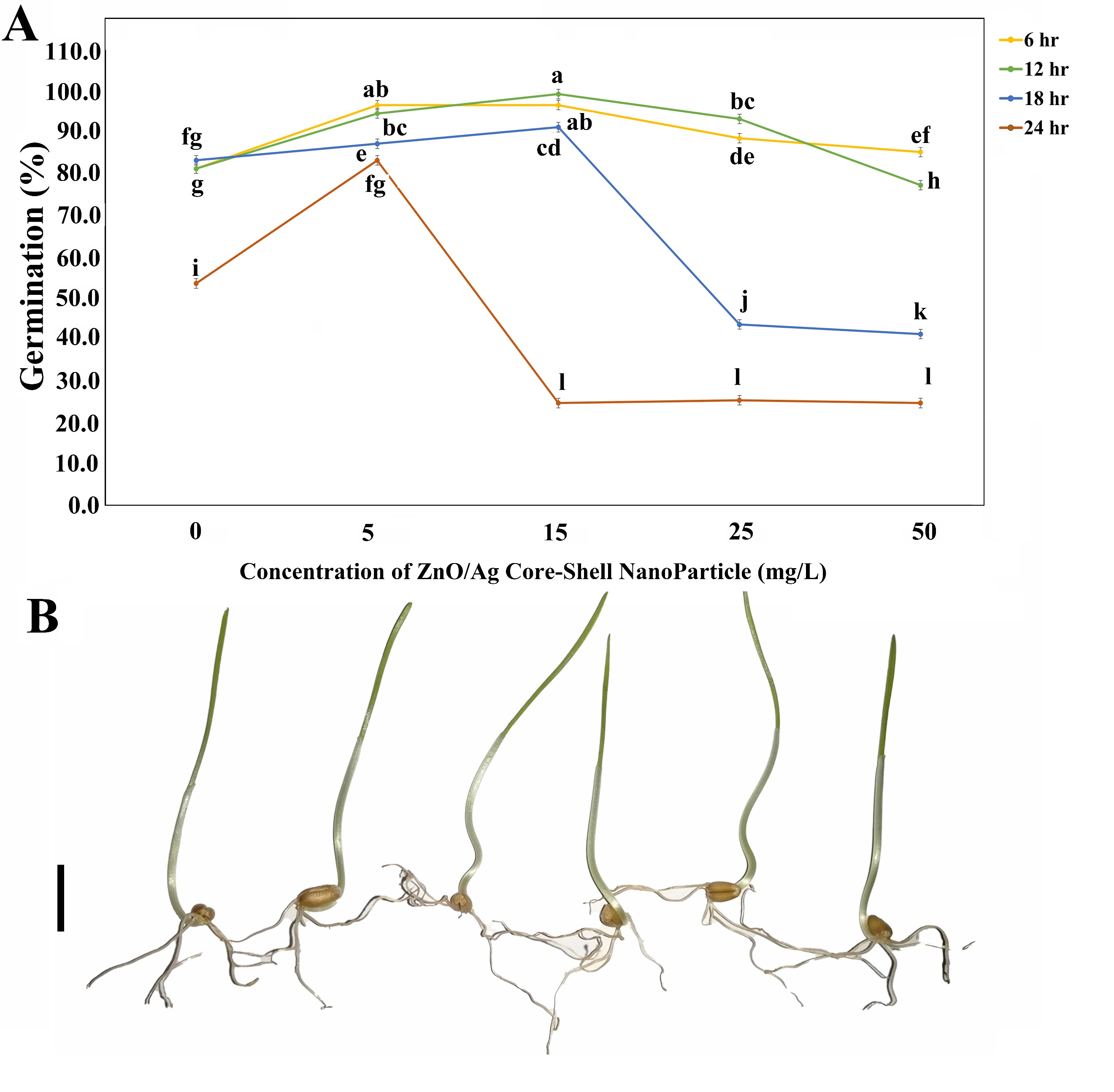
**

**Figure S3.** The effect of different priming treatments on seed germination percentage in wheat. (A) Effect of different priming treatments at different concentrations and different priming times, (B) Wheat seedlings hydroprimed with deionized water for 12 h. Significance was determined at p < 0.05 and the results are given as mean ± standard deviation. Different letters indicate significant differences among treatments.


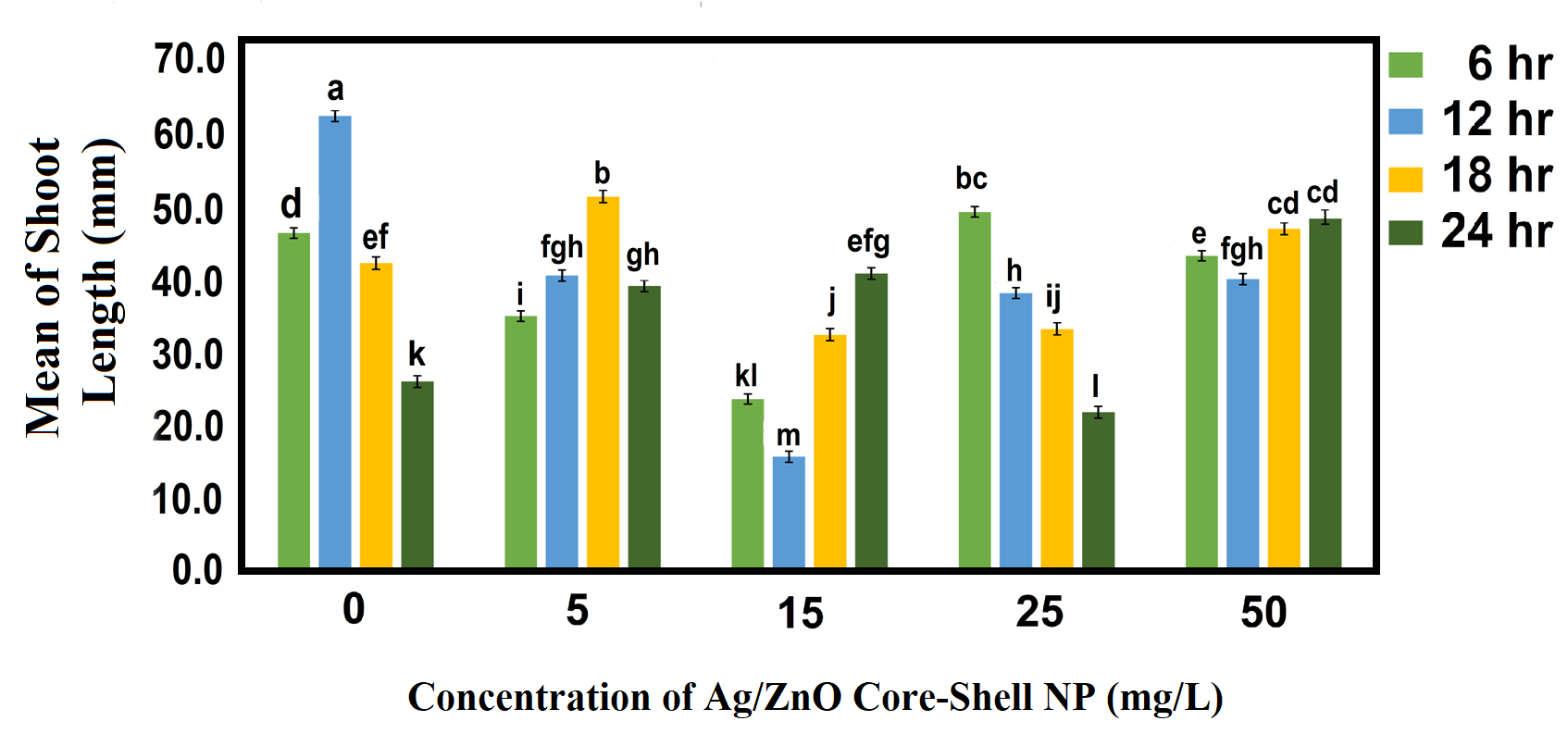


**Figure S4.** Impact of different priming treatments on the shoot length of wheat seeds. Significance was determined at p < 0.05 and the results are given as mean ± standard deviation. Different letters indicate significant differences among treatments.


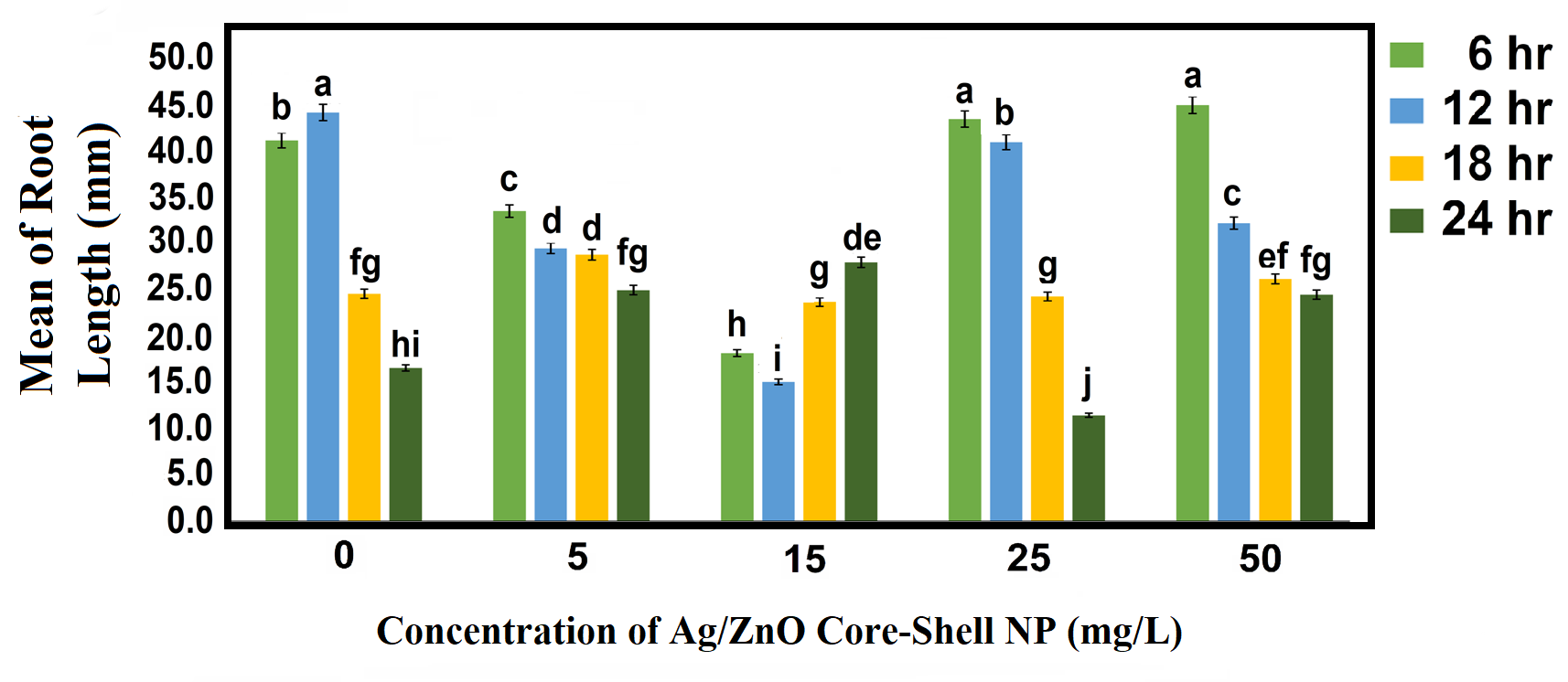


**Figure S5.** Impact of different priming treatments on the root length (RL) of wheat seeds. Significance was determined at p < 0.05 and the results are given as mean ± standard deviation. Different letters indicate significant differences among treatments.


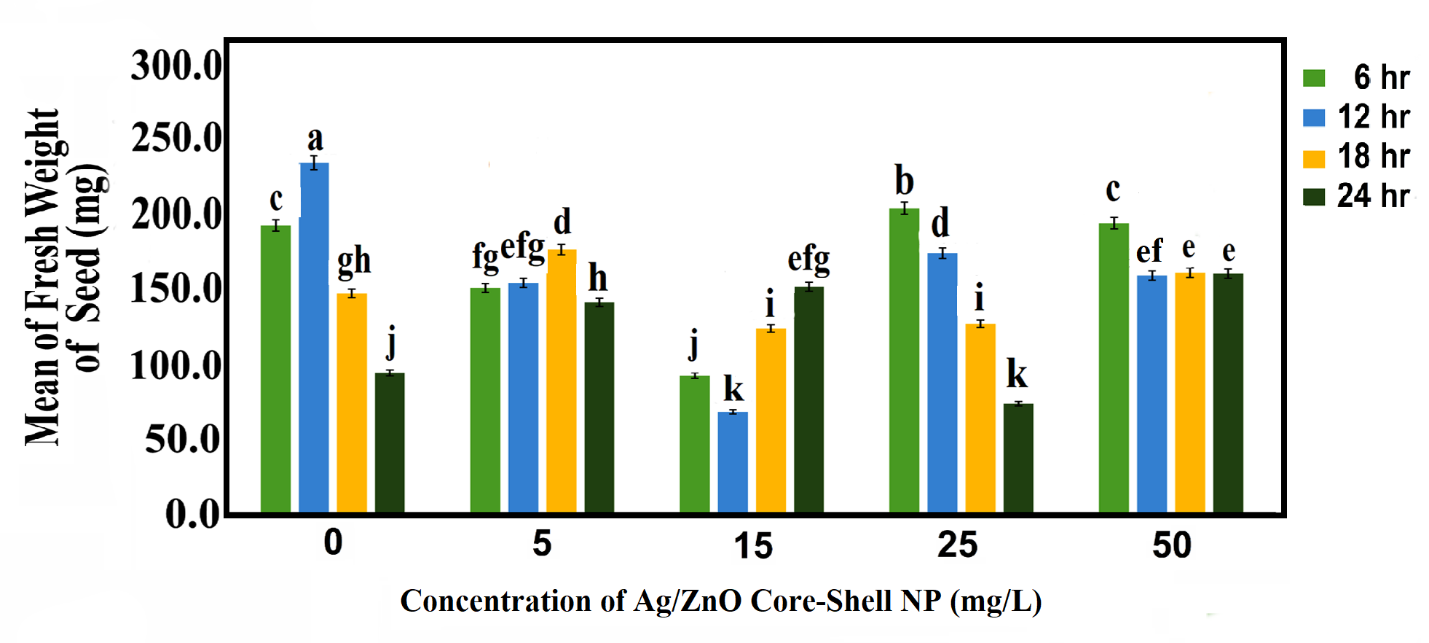


**Figure S6.** Impact of different priming treatments on the seedling fresh weight of wheat seeds. Significance was determined at p < 0.05 and the results are given as mean ± standard deviation. Different letters indicate significant differences among treatments.


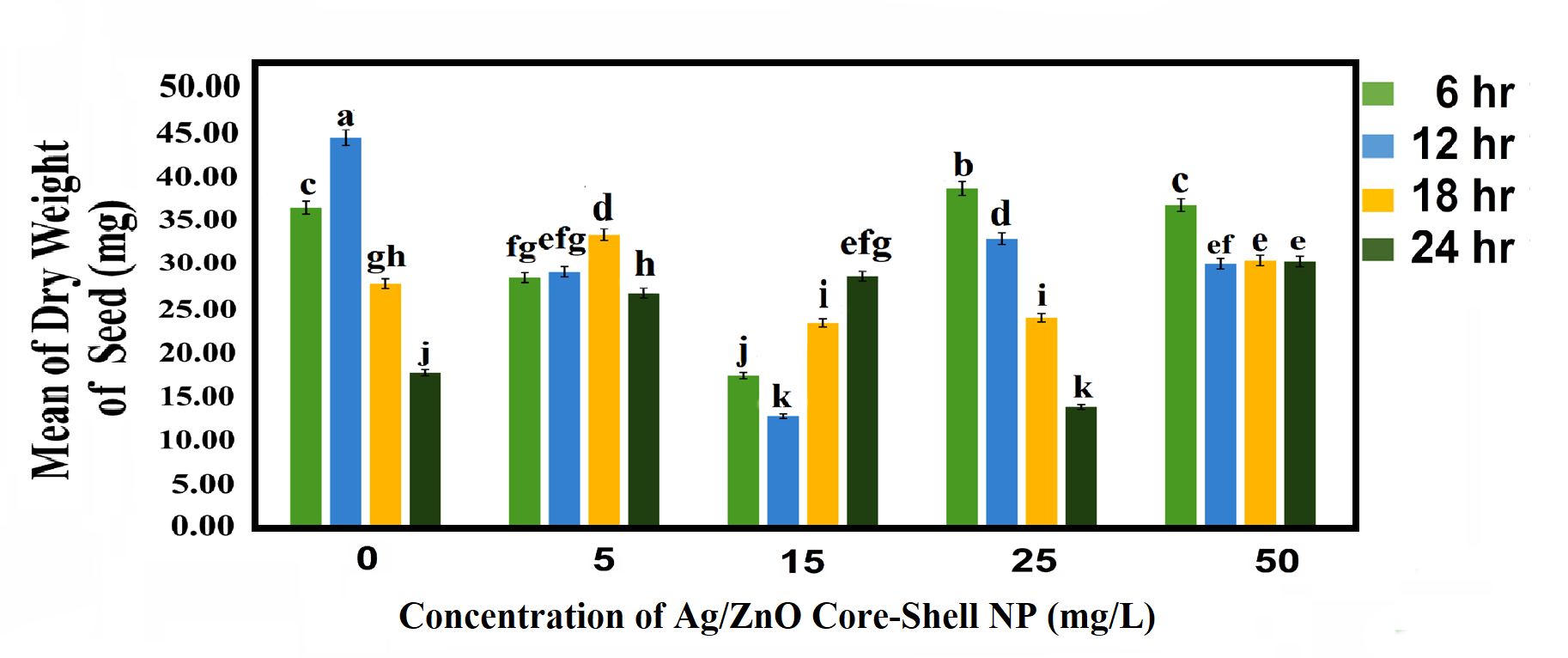


**Figure S7.** Impact of different priming treatments on the seedling dry weight of wheat seeds. Significance was determined at p < 0.05 and the results are given as mean ± standard deviation. Different letters indicate significant differences among treatments.


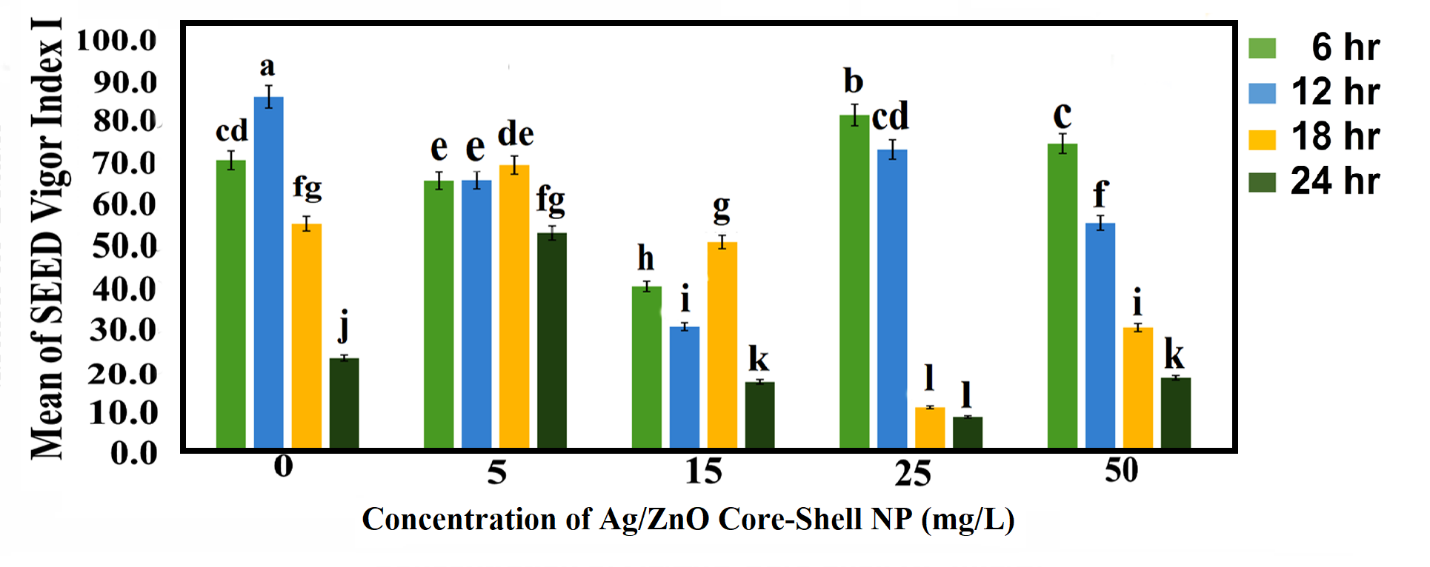


**Figure S8.** Impact of different priming treatments on the seed vigor index-I (SVI) of wheat seeds. Significance was determined at p < 0.05 and the results are given as mean ± standard deviation. Different letters indicate significant differences among treatments.


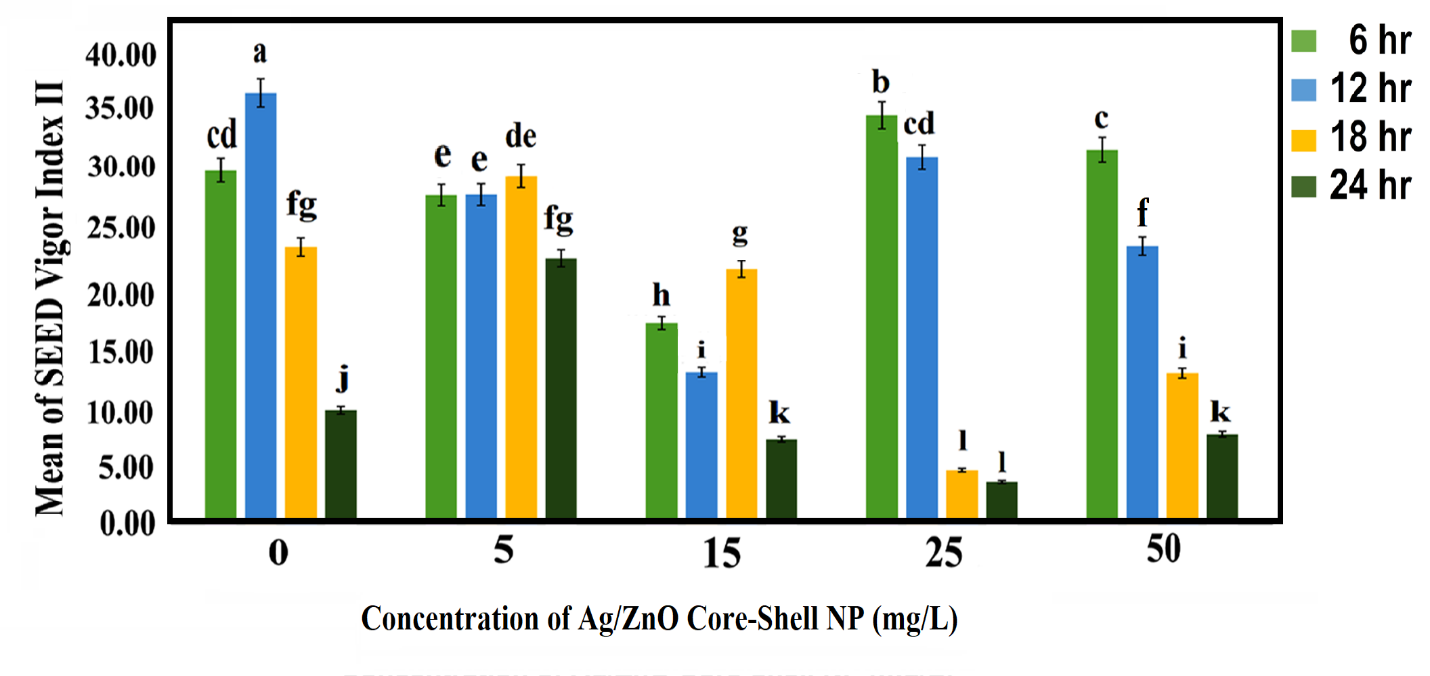


**Figure S9.** Impact of different priming treatments on the seed vigor index-II (SVII) of wheat seeds. Significance was determined at p < 0.05 and the results are given as mean ± standard deviation. Different letters indicate significant differences among treatments.


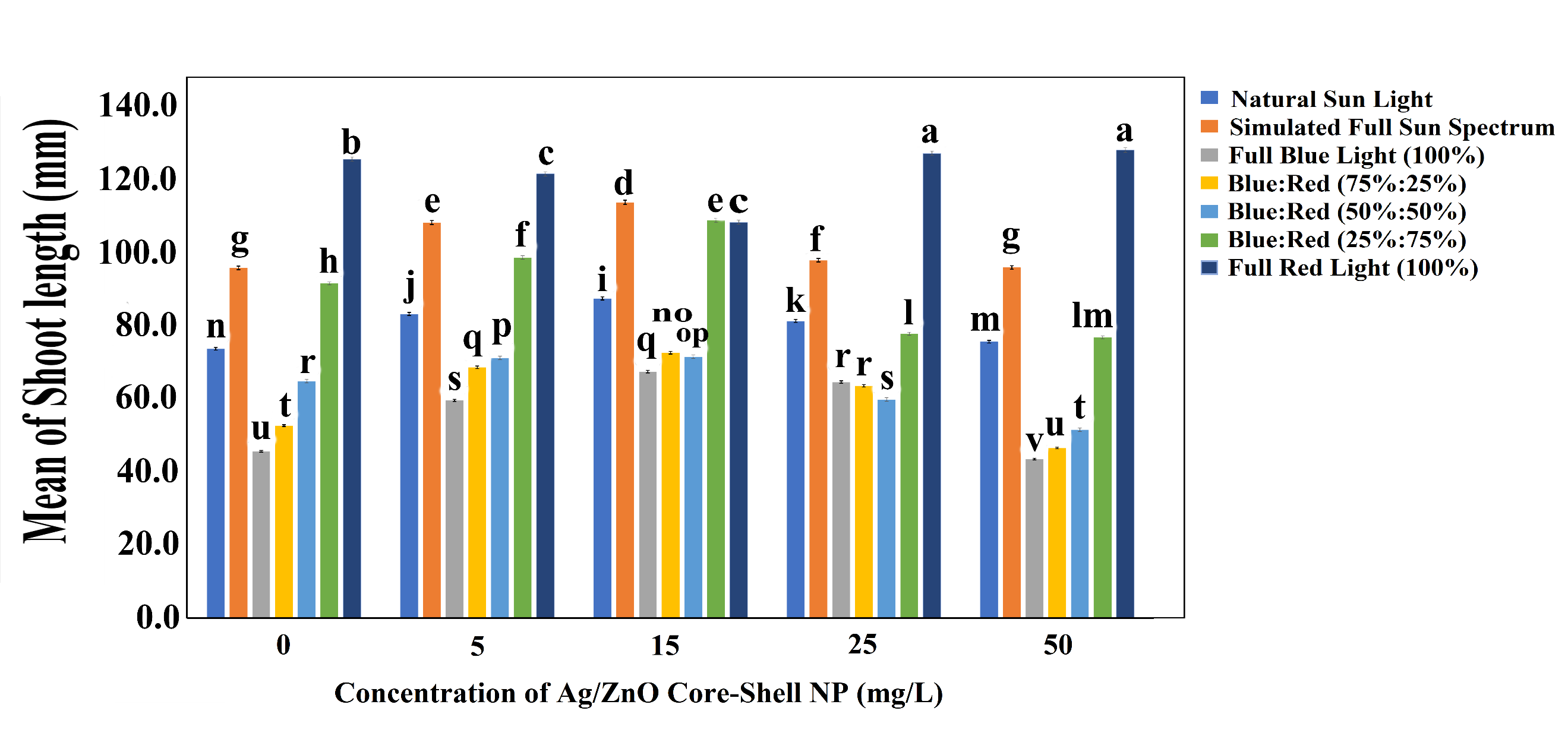


**Figure S10.** The means of shoot length (mm) in one-month-old wheat plants in different light conditions and Ag/ZnO NPs concentrations. The bars represent SD.


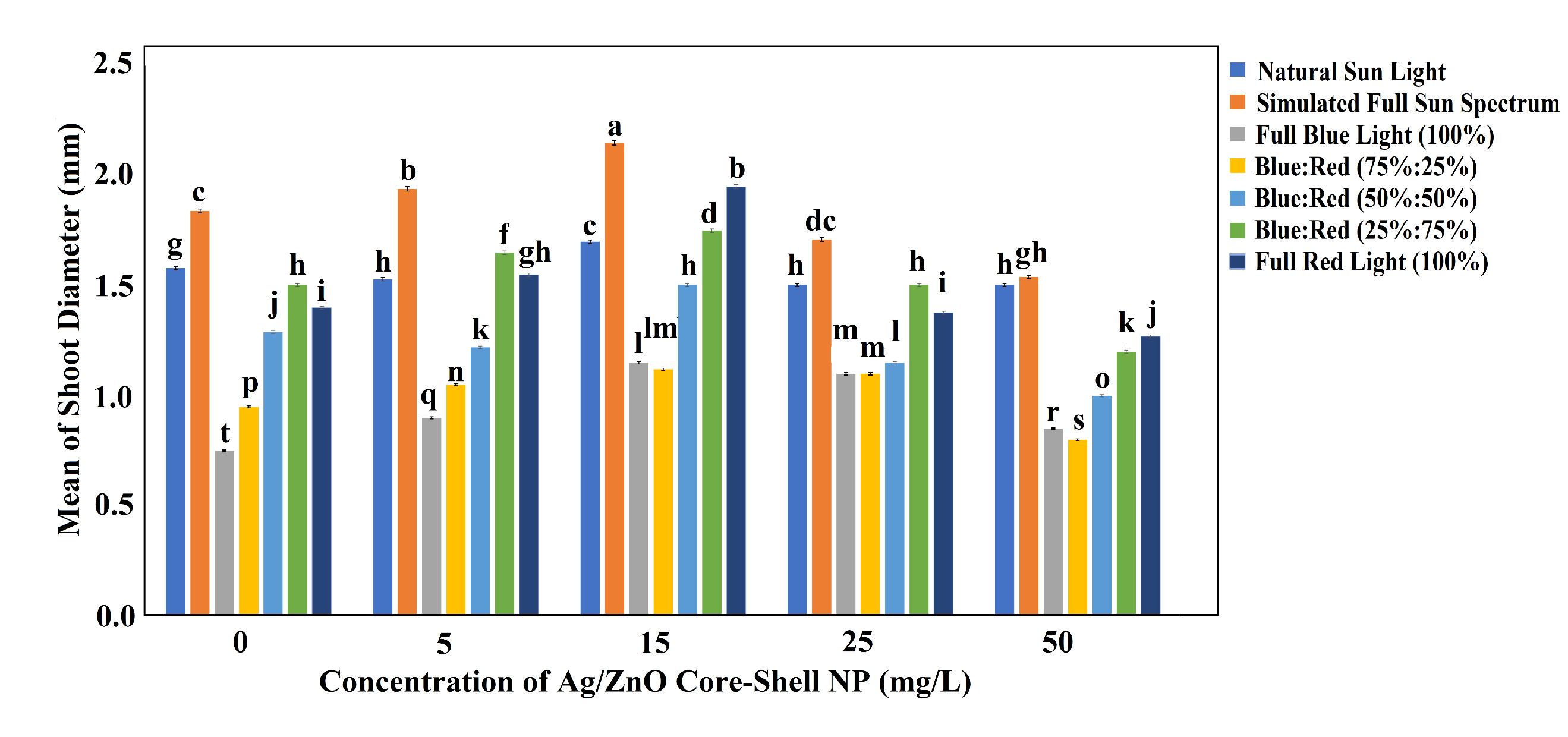


**Figure S11.** The means of Shoot diameter (mm) in one-month-old wheat plants in different light conditions and Ag/ZnO NPs concentrations. The bars represent SD.


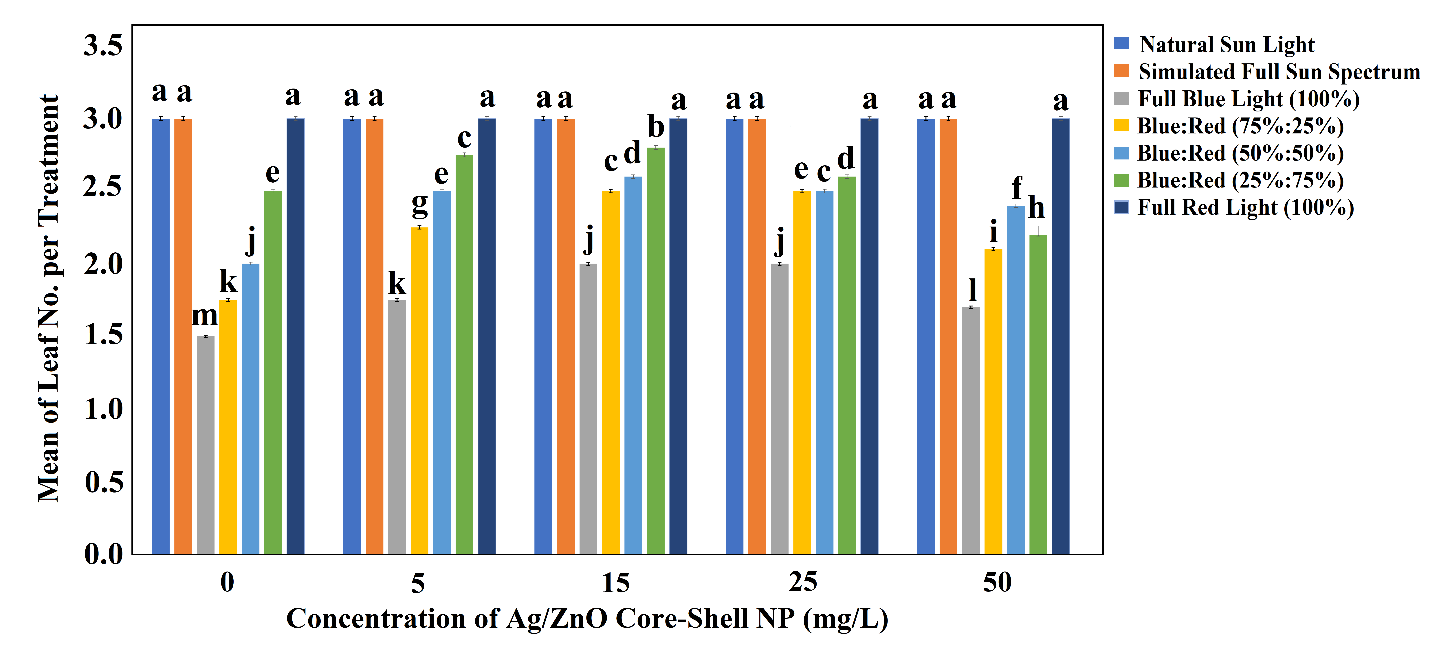


**Figure S12.** The means of Leaf number in one-month-old wheat plants in different light conditions and Ag/ZnO NPs concentrations. The bars represent SD.


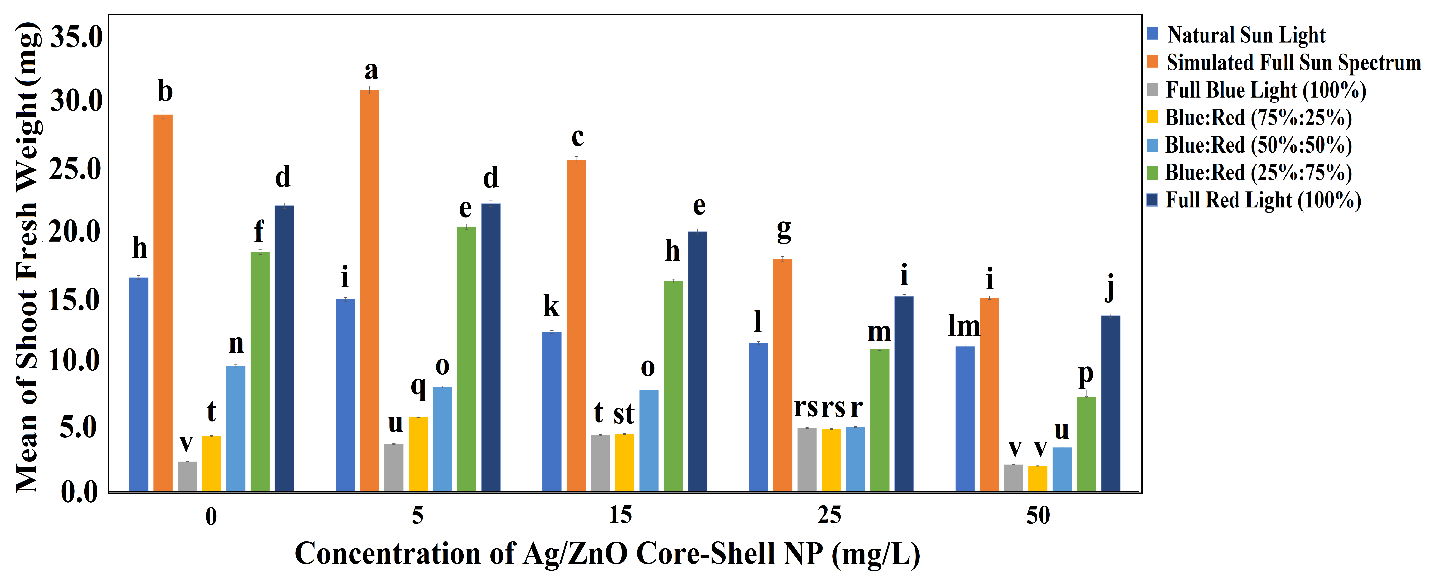


**Figure S13.** The means of shoot fresh weight (SFW) (mg) in one-month-old wheat plants in different light conditions and Ag/ZnO NPs concentrations. The bars represent SD.


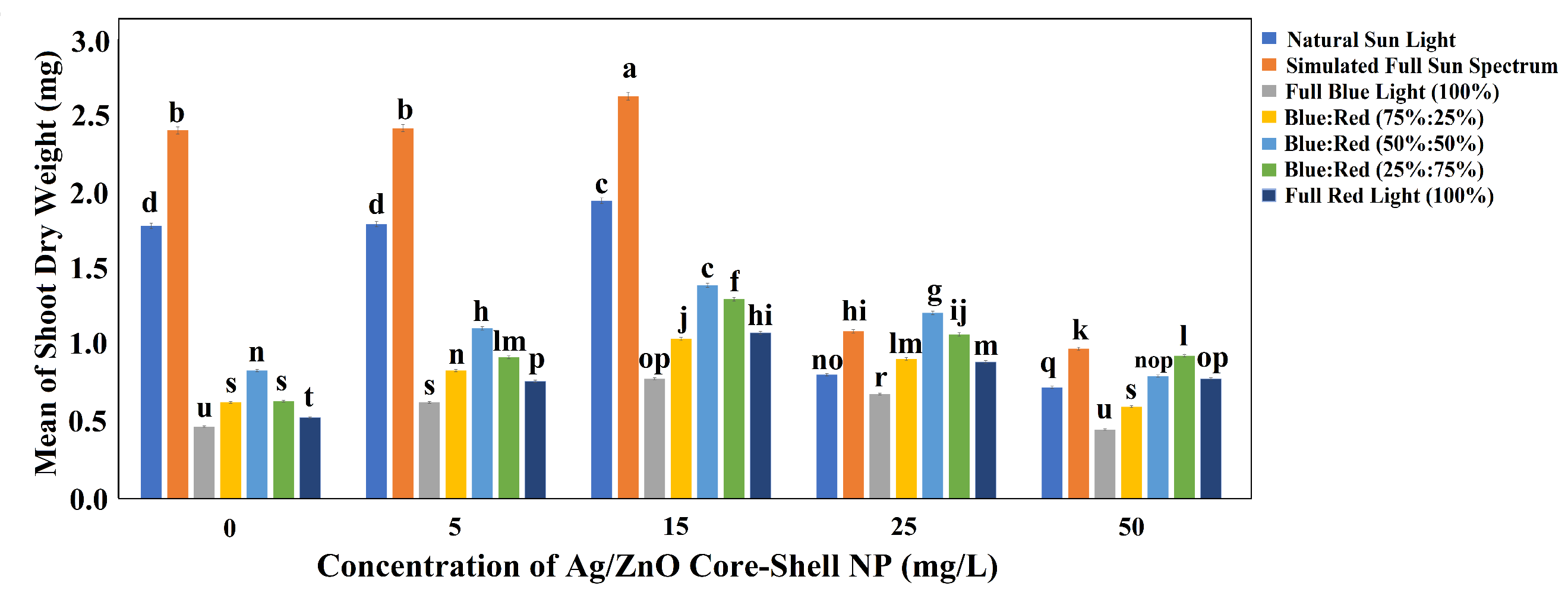


**Figure S14.** The means of shoot dry weight (SDW) (mg) in one-month-old wheat plants in different light conditions and Ag/ZnO NPs concentrations. The bars represent SD.


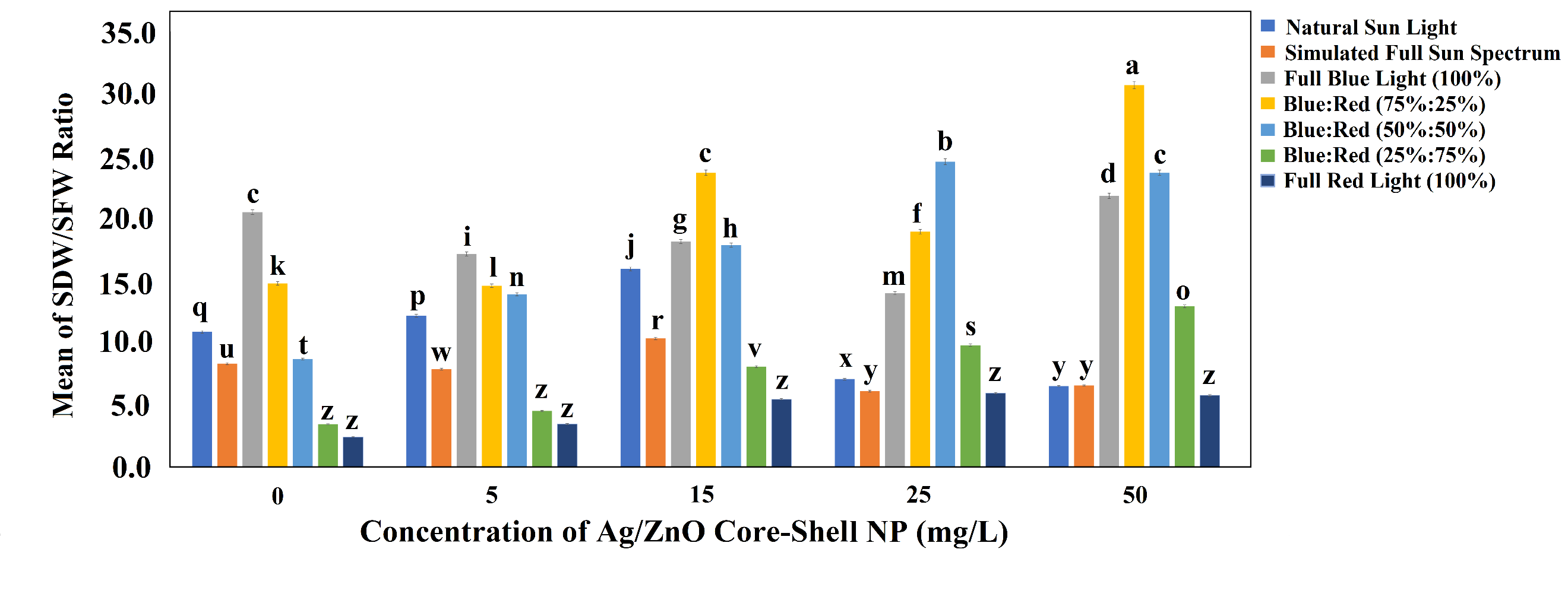


**Figure S15.** The means of the ratio of SDW to SFW in one-month-old wheat plants in different light conditions and Ag/ZnO NPs concentrations. The bars represent SD.


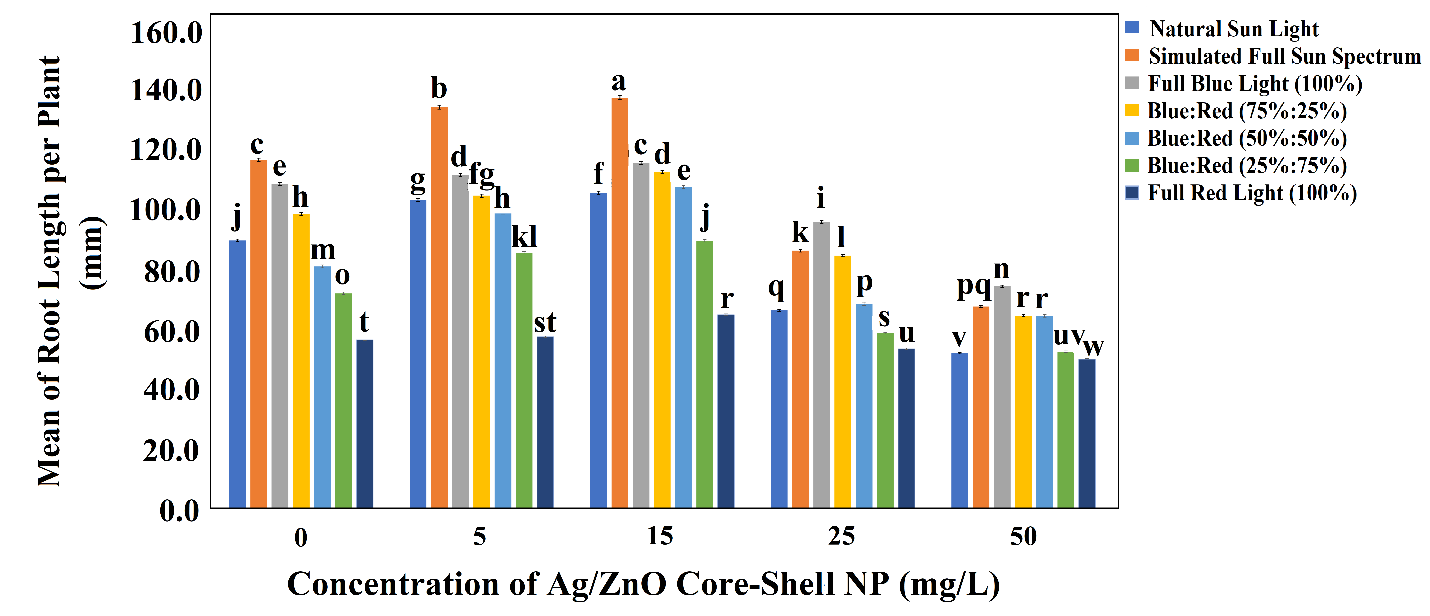


**Figure S16.** The means of Root length (mm) in one-month-old wheat plants in different light conditions and Ag/ZnO NPs concentrations. The bars represent SD.


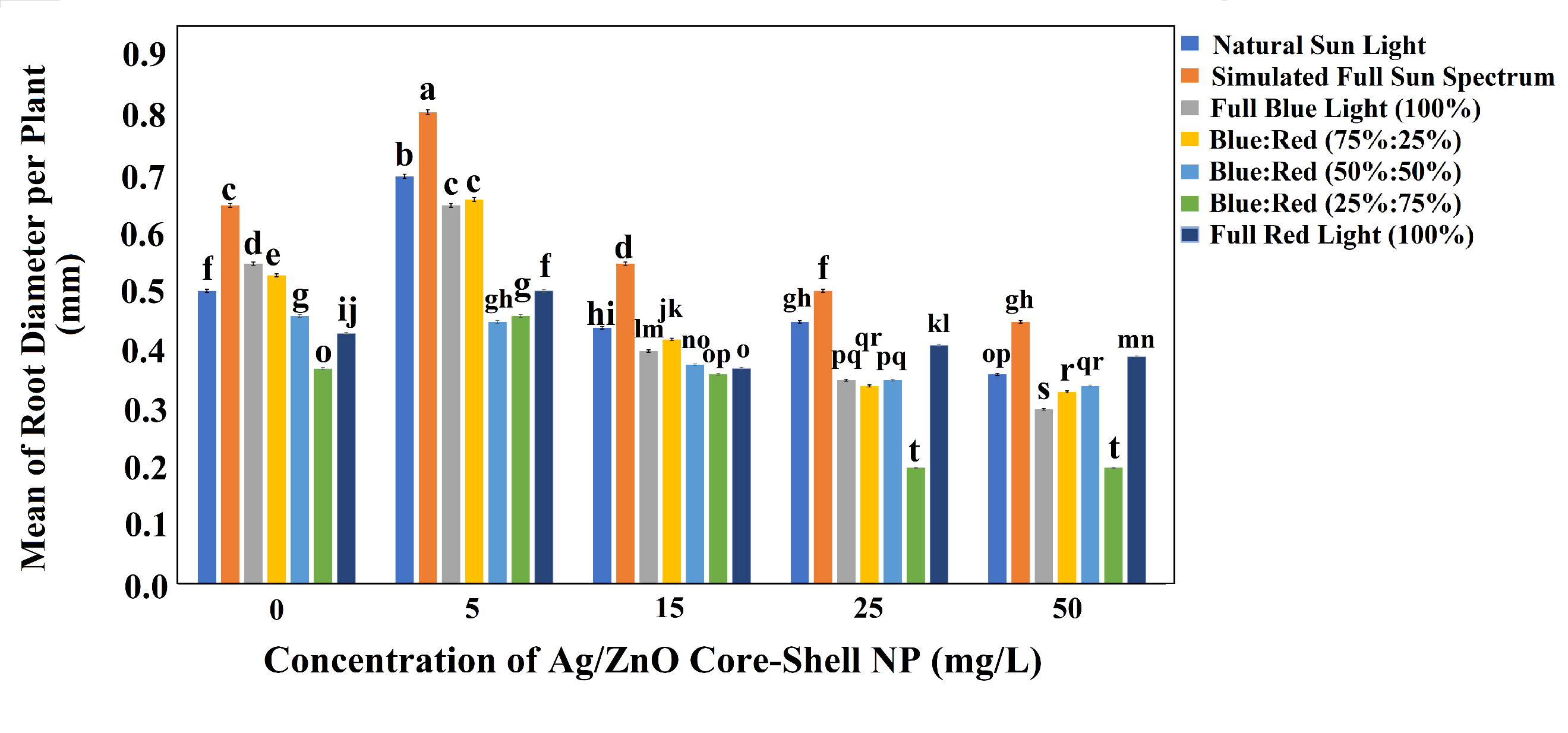


**Figure S17.** The means of Root diameter (mm) in one-month-old wheat plants in different light conditions and Ag/ZnO NPs concentrations. The bars represent SD.


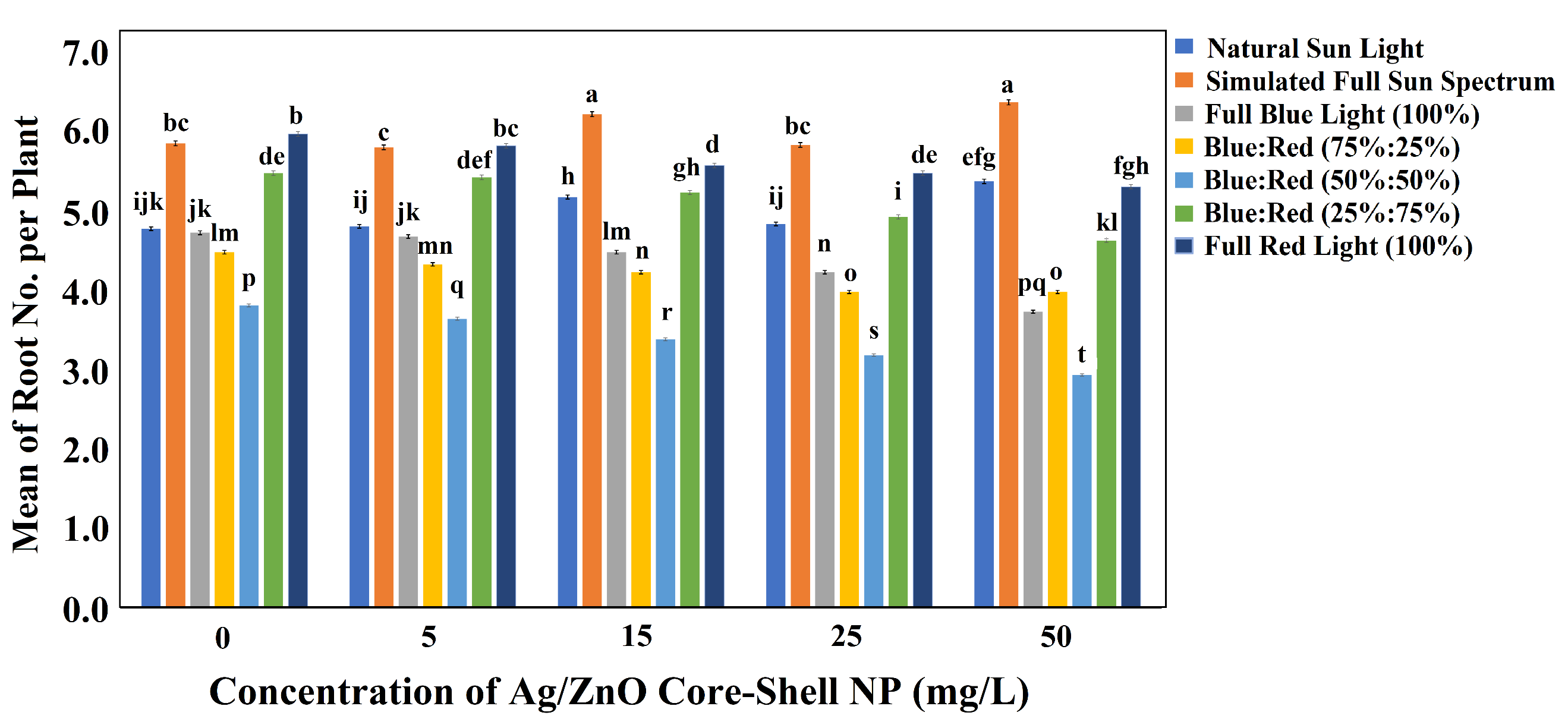


**Figure S18.** The means of number of roots in one-month-old wheat plants in different light conditions and Ag/ZnO NPs concentrations. The bars represent SD.


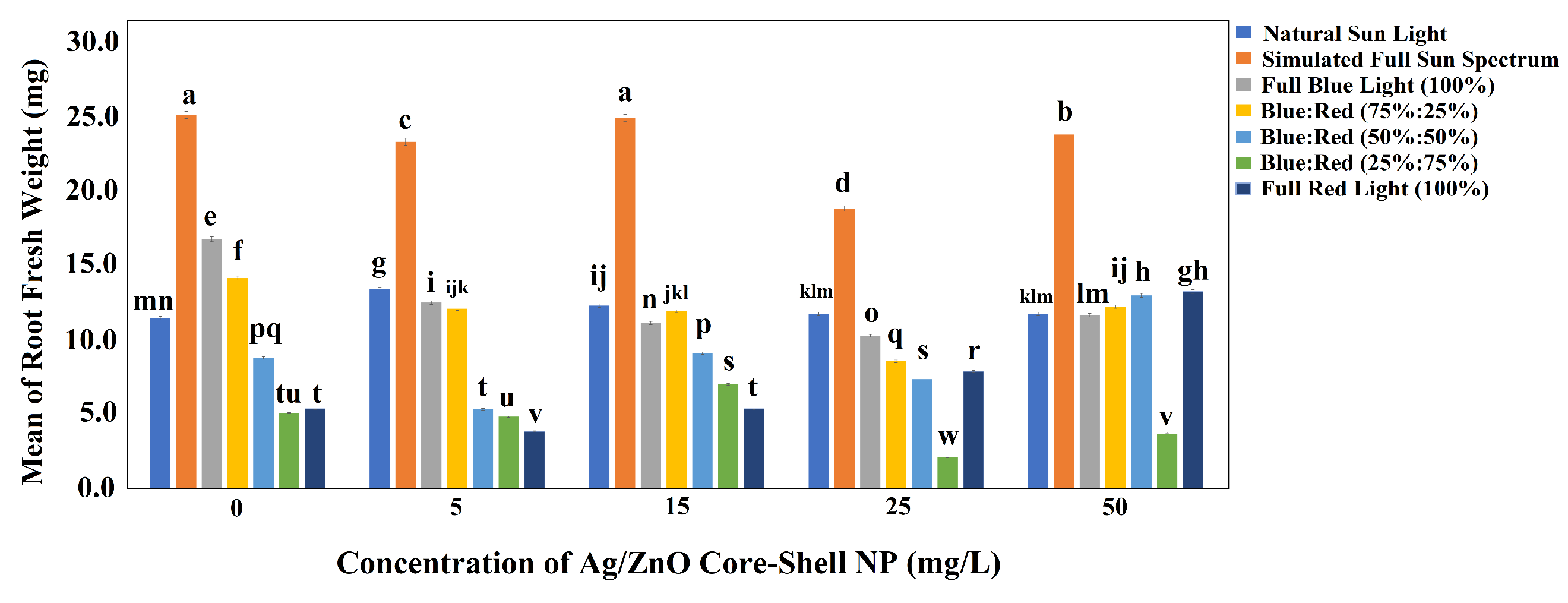


**Figure S19.** The means of Root fresh weight (RFW) (mg) in one-month-old wheat plants in different light conditions and Ag/ZnO NPs concentrations. The bars represent SD.


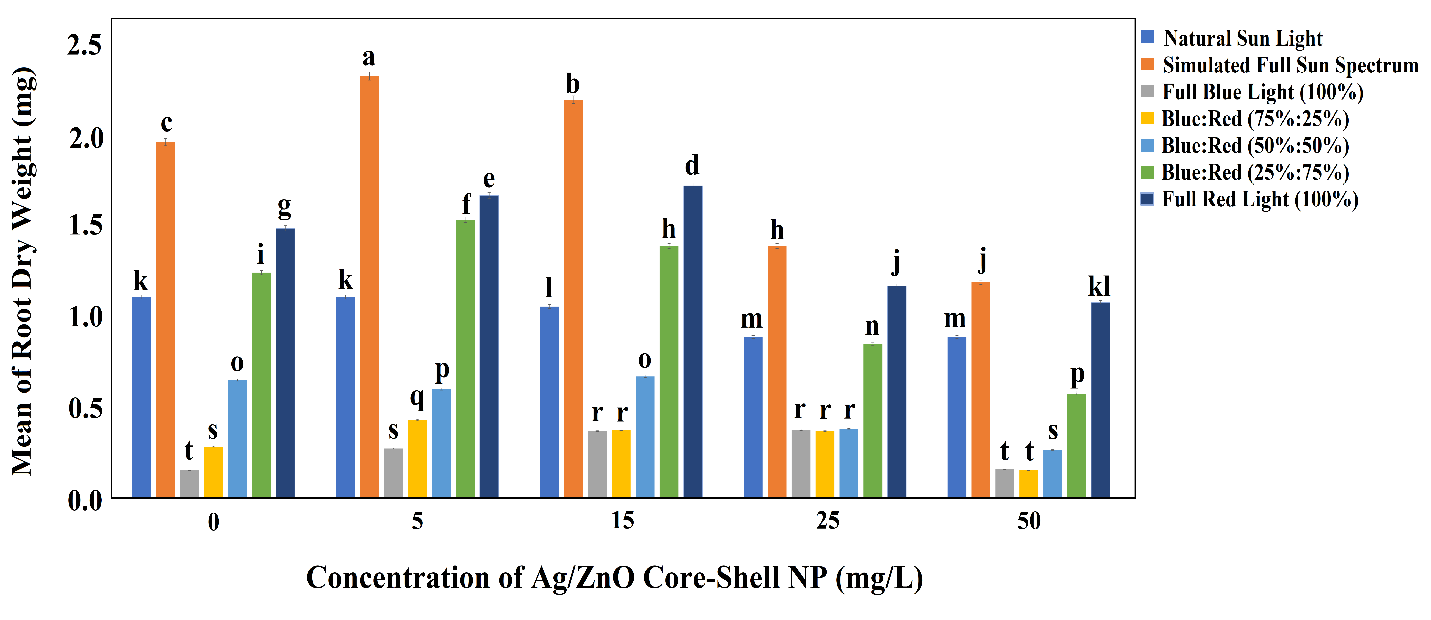


**Figure S20.** The means of Root dry weight (RDW) (mg) in one-month-old wheat plants in different light conditions and Ag/ZnO NPs concentrations.The bars represent SD.


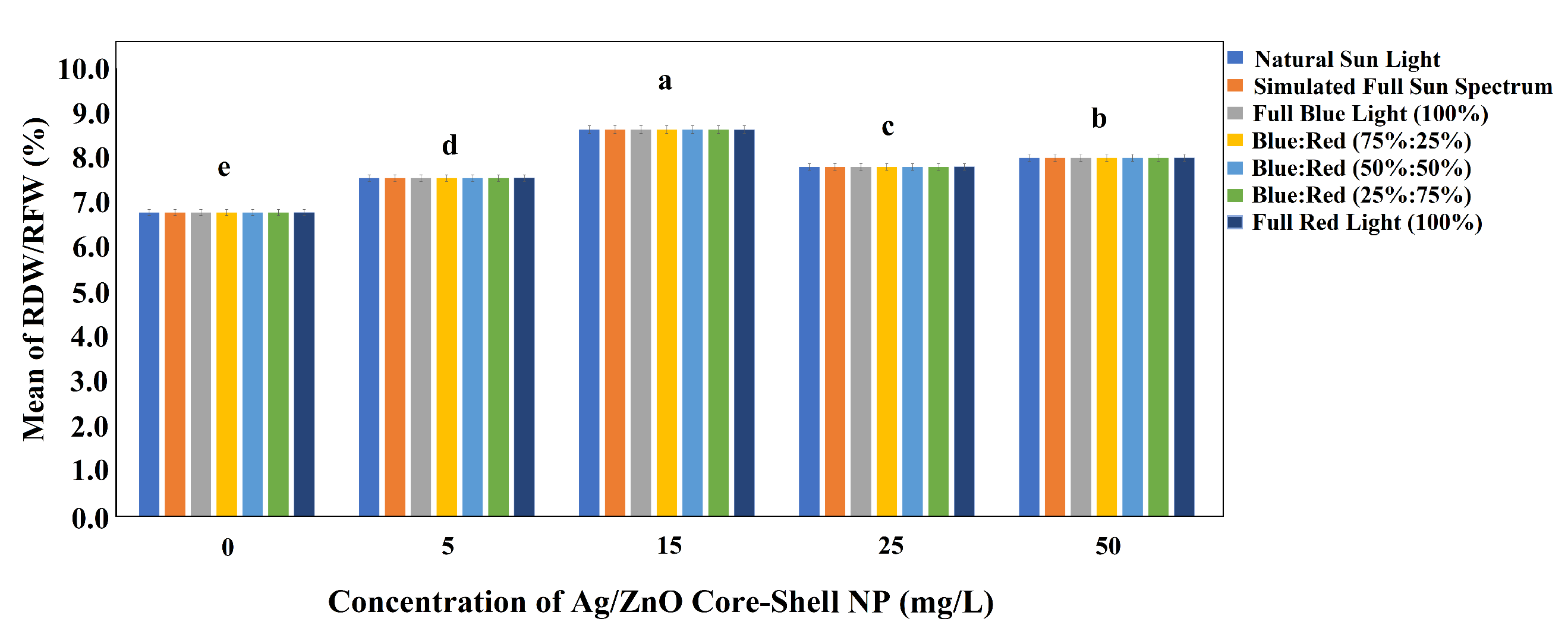


**Figure S21.** The means of the ratio of RDW to RFW in one-month-old wheat plants in different light conditions and Ag/ZnO NPs concentrations. The bars represent SD.

**
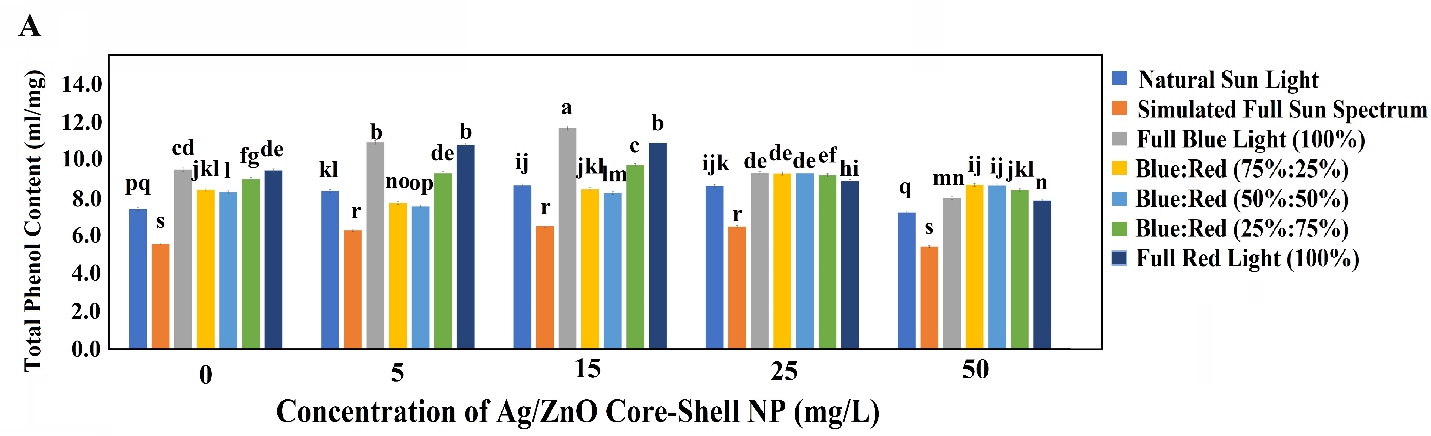
**

**Figure S22.** The means of total phenol content (ml/mg) in one-month-old wheat plants in different light conditions and Ag/ZnO NPs concentrations. The bars represent SD.


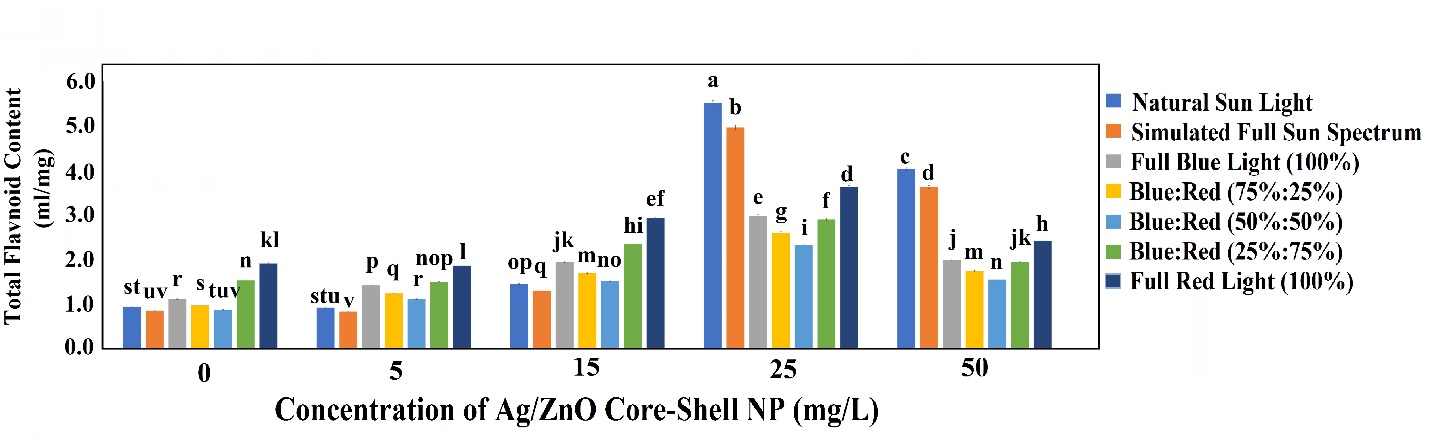


**Figure S23.** The means of total flavonoids content (ml/mg) in one-month-old wheat plants in different light conditions and Ag/ZnO NPs concentrations. The bars represent SD.


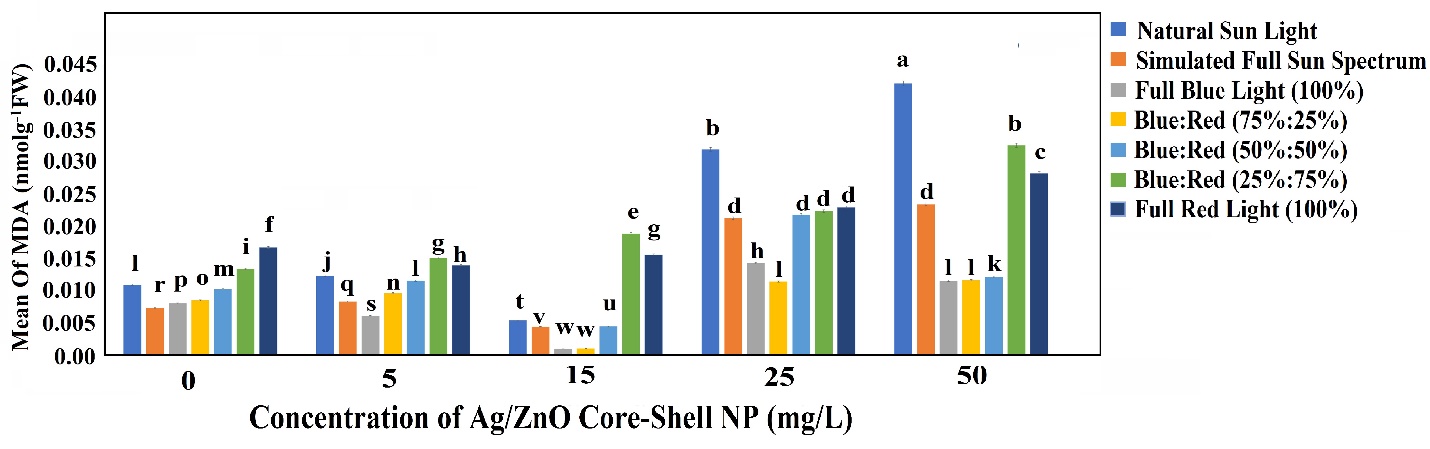


**Figure S24.** The means of total lipid peroxidation (nmol g^-1^ FW) in one-month-old wheat plants in different light conditions and Ag/ZnO NPs concentrations. The bars represent SD.
